# Major influencing factors on routine implementation of shared decision-making in cancer care: qualitative process evaluation of a stepped-wedge cluster randomized trial

**DOI:** 10.1186/s12913-023-09778-w

**Published:** 2023-08-08

**Authors:** Pola Hahlweg, Anja Lindig, Wiebke Frerichs, Jördis Zill, Henning Hanken, Volkmar Müller, Mia-Carlotta Peters, Isabelle Scholl

**Affiliations:** 1 Department of Medical Psychology, University Medical Center Hamburg-Eppendorf, Martinistr. 52, 20246 Hamburg, Germany; 2 Center of Health Care Research, University Medical Center Hamburg-Eppendorf, Martinistraße 52, 20246 Hamburg, Germany; 3 Department of Oral, Maxillofacial and Plastic Surgery, Asklepios Klinik Nord – Heidberg, Tangstedter Landstr. 400, 22417 Hamburg, Germany; 4 Department of Gynecology, University Medical Center Hamburg-Eppendorf, Martinistr. 52, 20246 Hamburg, Germany; 5 II. Department of Medicine, University Medical Center Hamburg-Eppendorf, Martinistr. 52, 20246 Hamburg, Germany

**Keywords:** Shared decision-making, Implementation science, Consolidated Framework for Implementation Research, Process evaluation, Qualitative methods, Oncology

## Abstract

**Background:**

Shared decision-making (SDM) is highly relevant in oncology but rarely implemented in routine care. In a stepped-wedge cluster randomized implementation trial, the outcome evaluation of a theoretically and empirically based multi-component SDM implementation program did not show a statistically significant effect on patient-reported SDM uptake. Within this SDM implementation trial, a thorough a priori planned process evaluation was conducted. Thus, the aim of this study was to investigate factors influencing SDM implementation in the context of a multi-component SDM implementation program.

**Methods:**

We conducted qualitative process evaluation of a stepped-wedge SDM implementation trial. Qualitative data included interviews with nurses and physicians of participating departments, field notes by the study team, and meeting minutes. Data were analyzed via deductive and inductive qualitative content analysis on basis of the Consolidated Framework for Implementation Research (CFIR).

**Results:**

Transcripts of 107 interviews with 126 nurses and physicians, 304 pages of field note documentation, and 125 pages of meeting minutes were analyzed. Major factors influencing SDM implementation were found for all domains of the CFIR: a) four regarding characteristics of the individuals involved (e.g., perceived personal relevance, individual motivation to change), b) eleven regarding the inner setting (e.g., leadership engagement, networks and communication, available resources, compatibility with clinical practice), c) two regarding the outer setting (e.g., culture of health care delivery), d) eight regarding characteristics of the intervention (e.g., relative advantage, adaptability), and e) three regarding the implementation process (e.g., integration into existing structures). Furthermore, we found strong interrelations between several of the influencing factors within and between domains.

**Conclusions:**

This comprehensive process evaluation complements the outcome evaluation of the SDM implementation trial and adds to its interpretation. The identified influencing factors can be used for planning, conducting, and evaluating SDM implementation in the future.

**Trial registration:**

clinicaltrials.gov, NCT03393351, registered 8 January 2018, https://clinicaltrials.gov/ct2/show/NCT03393351

**Supplementary Information:**

The online version contains supplementary material available at 10.1186/s12913-023-09778-w.

## Background

Shared decision-making (SDM), a medical decision-making process between patients and health care professionals (HCP) on an equal footing [1–3], is seen as pivotal to patient-centered health care and evidence-based medicine [4, 5]. In cancer care, where many complex treatment options with considerable effects on patients’ quality of life exist, SDM has been argued to be especially important [6–8]. However, uptake of SDM in routine care continues to lag behind [9–13]. SDM is the intervention being examined in this study.

Influencing factors on the uptake of SDM in routine care have been examined on different levels. A systematic review assessing barriers and facilitators on the individual HCP level found limited time and SDM not being applicable due to patient characteristics or due to the clinical situation to be the most frequently reported barriers to SDM implementation [14]. Motivated HCPs and expected positive impact of SDM on the process or patient outcomes were the most often found facilitators for SDM [14]. Similar influencing factors were also seen in a pre-implementation phase preceding this trial [10, 11, 15]. Within a scoping review investigating influencing factors on SDM implementation on the organizational and health system level, factors such as leadership engagement, organizational culture, relative priorities, and teamwork were found on the organizational level [16]. On the health system level, policies and guidelines, incentivization, learning opportunities, and licensing were found to influence SDM implementation [16]. A large-scale multi-component SDM implementation program in the UK reported similar influencing factors [17].

Multi-component implementation programs (also called complex interventions, [18]) have been on the rise to foster SDM implementation. In national and international routine care, those trials were recently undertaken in the UK [17], Germany [19, 20], and the Netherlands [21]. The present study was conducted as part of a cluster-randomized trial following a stepped wedge design to evaluate a theoretically and empirically based multi-component SDM implementation program [20, 22].

Implementation science advises to base trials evaluating implementation programs on theory [23, 24]. The Consolidated Framework for Implementation Research (CFIR, [25]) offers such a theoretical underpinning. The CFIR includes five major domains, the individual, the intervention, the inner setting (i.e., organizational level), the outer setting (i.e., system level), and the implementation process, and 39 subordinate constructs to those domains. Hence, the CFIR offers a comprehensive framework to develop and evaluate implementation efforts. In addition, the Medical Research Councils guidance on multi-faceted implementation programs calls for a combination of outcomes and process evaluation [26]. A thorough process evaluation can help to recognize implementation problems and explain effects (or lack thereof) in the outcomes evaluation [27, 28].

Thus, the aim of this study was to investigate influencing factors on SDM implementation in the context of a theoretically and empirically based multi-component SDM implementation program by means of an a priori planned process evaluation. The exploration of these influencing factors complemented the outcomes evaluation of a cluster-randomized implementation trial to foster SDM in routine cancer care and added to the understanding of its results [20].

## Methods

### Study design

This is a qualitative study describing the process evaluation within the cluster-randomized implementation trial “Evaluation of a program for routine implementation of shared decision-making in cancer care: a stepped wedge cluster randomized trial” (PREPARED) [20, 22]. The process evaluation examines influencing factors on SDM implementation in general and on the six implementation strategies used in this trial. It includes qualitative data from the perspectives of participating HCPs’ (physicians and nurses) and the study team. For presenting the process evaluation, we followed the consolidated criteria for reporting qualitative research checklist (COREQ, [29], see Additional File 1).

### Overview of the underlying cluster-randomized SDM implementation trial

**Fig. 1:**
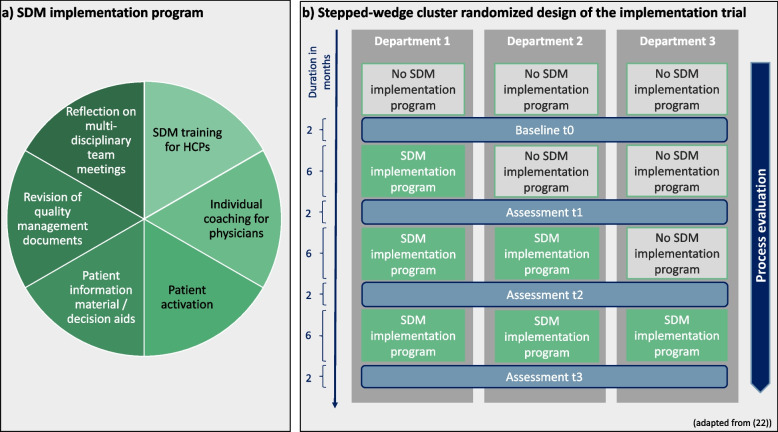
Overview of the cluster-randomized SDM implementation trial

The cluster-randomized PREPARED trial followed a stepped wedge design to evaluate a theoretically and empirically based multi-component SDM implementation program [20, 22]. Details on methodology of the entire PREPARED trial can be found in the study protocol [22]. The outcome evaluation of the trial was summarized below and reported elsewhere in detail [20]. For a depiction of the implementation program and the trial design see Fig. 1.

The SDM implementation program was implemented at three departments (clusters) of the University Cancer Center Hamburg (UCCH), a comprehensive cancer center at the University Medical Center Hamburg-Eppendorf (UKE) in Germany. It consisted of six implementation strategies: 1) SDM group training for physicians and nurses, 2) individual coaching sessions for physicians, 3) provision of the patient empowerment strategy Ask 3 Questions (Ask3Q, [30, 31]), 4) provision of patient information materials and a generic patient decision aid [32, 33], 5) integration of SDM into the department’s quality management documents, and 6) reflection meetings with responsible clinical stakeholders on the integration of SDM in multidisciplinary team meetings (MDTMs). SDM training in this implementation program was planned following a train-the-trainer approach. A small group of physicians and nurses (multipliers) received a five-hour SDM training. Together with the research team, those multipliers were expected to offer SDM team training sessions to their colleagues afterwards. While all implementation strategies were implemented in all departments, adaptations occurred (e.g., reach and coaching dose per person were lower than planned, trainings were less interdisciplinary) [20].

The collection of outcome data occurred in four two-months waves before, between, and after the implementation intervals (cp. Fig. 1) [20]. Patient-reported uptake of SDM measured by the 9-item Shared Decision Making Questionnaire (SDM-Q-9, [34]) was the primary endpoint [20]. However, despite the thorough execution and evaluation of the trial, the outcome evaluation did not show statistically significant differences in the primary endpoint and most secondary outcomes before and after implementation of the multi-component SDM implementation program [20].

### Data collection

Qualitative process evaluation data was collected systematically throughout the trial. The implementation program was sequentially introduced to the participating departments in a randomized order through three implementation intervals with a duration of six months each (cp. Fig. 1). Process interview data was collected during the three implementation intervals of the SDM implementation trial as soon as implementation in this department had started. A purposive sampling with maximum variation approach (i.e. theoretical sampling led by the purpose of the evaluation seeking a sample with maximum heterogeneity, [35]) was used for physicians and nurses who worked on inpatient wards and at outpatient clinics of the participating departments. The number of interviews per department was specified a priori dependent on the size of the department. For each implementation interval, we planned to conduct interviews with three people per ward/clinic at each department that was receiving the SDM program. The number of interviews was defined by the study team a priori and was a trade-off between the assumption that pragmatic data saturation could be reached and feasibility [36]. To conduct interviews, a member of the study team (cp. researchers’ characteristics below) visited the wards and clinics of the participating departments and invited HCPs to take part in a short interview. Interviewees could be physicians or nurses. Following the cluster-randomized design, we invited HCPs in the respective departments to be interviewed irrespective of individual prior participation in the implementation strategies. If they agreed to participate, interviews were either conducted on the spot or an appointment for an interview was made. Process interviewers were not involved in executing the SDM implementation program or in assessing quantitative outcome evaluation data of the trial, in order to disconnect process evaluation data collection and facilitate straightforward answers from HCPs. There were no relationships between process interviewers and participants established prior to study commencement. Due to the data collection setting (e.g., in ward rooms), it was possible that people not participating in the interview were in the same room during interviews.

Qualitative process interviews with HCPs followed a semi-structured interview guide, which was not pilot tested. Interviews were conducted to assess HCPs’ views on SDM implementation in general and the six implementation strategies of the implementation program in particular. Participants were asked about 1) their knowledge of the trial, the implementation program and its six implementation strategies, 2) their access to knowledge and information about the trial and implementation program, 3) their experience when participating in SDM group training and individual coaching sessions, 4) their attitudes and beliefs about the implementation strategies including influencing factors on their implementation, and 5) additional feedback regarding the trial, implementation program, or SDM in general. Additionally, demographic data (i.e., age, profession, work experience) were assessed from all participants. The interview guide can be found in Additional File 2. Interviews were audio-recorded and transcribed verbatim. No additional field notes were conducted. For one interview, the participant refused the audio-recording and a written protocol of the interview was conducted instead. Transcripts and the protocol were not returned to participants for comments and/or corrections and participants did not provide feedback on findings.

We additionally collected qualitative observational data through field notes during the preparation phase and all implementation intervals and measurement waves of the trial [37]. All members of the study team included their observations at the participating departments and experiences with cooperators and participants in these field notes. Field notes during implementation intervals could e.g., include a SDM trainer’s or coach’s perception of participants’ interest and motivation in SDM implementation or a study team member’s conversation in passing with a cooperation partner about one of the implementation strategies.

Last, minutes of meetings with clinical cooperators and of SDM training sessions were included in the qualitative process evaluation. However, minutes did not exist for every meeting or training session due to limited personnel resources.

### Data analysis

Data was analyzed using qualitative content analysis as described by Hsieh and Shannon [38] to investigate influencing factors on SDM implementation in general and on the implementation strategies used in this SDM implementation program in particular. We used a predominantly deductive approach, but remained open for inductive additions to our coding scheme. Deductive codes were predefined according to the domains and constructs of the CFIR [25] and a scoping review on organizational and system-level characteristics that influence implementation of SDM [16]. All factors of the CFIR were included in the initial deductive coding scheme. For every coded passage, additional meta-codes were allocated describing the department (i.e., department 1, 2, or 3), the direction of the influencing factor (i.e., barrier or facilitator), and whether the coding concerned SDM in general or one of the implementation strategies in particular (i.e., SDM in general, SDM group training, coaching, Ask3Q, patient information material, quality management documents, MDTMs).

Data analysis was conducted as follows: 1) Creation of a coding scheme with deductive codes (PH), 2) meeting to review the coding scheme with the research team (PH, IS, HC (cp. acknowledgements)), 3) coding of approximately 25% of the data (including transcripts, field notes, and minutes) by PH, 4) coding of another approximately 25% of the data (including transcripts, field notes, and minutes) by AL, 5) discussion of ambiguities and open questions between PH and AL and revision of the coding scheme, 6) coding of the remaining data by AL, 7) discussion and revision of codings and coding scheme between PH and AL until consensus was reached, 8) comprehensive quality control (i.e., PH reviewed all codings initially assigned by AL and vice versa), 9) discussion of ambiguities and open questions between PH and AL and revision of codings and coding scheme, 10) meeting to review the coding scheme with the research team (PH, AL, IS, HC), 11) revision and finalization of the coding scheme and the codings by PH and AL.

After the coding scheme was finalized, PH and AL independently classified each code as a major, mid-level, minor, or no influencing factor within this SDM implementation trial. The results were discussed by PH and AL until consensus was reached for each code. In the main body of this manuscript, we present only codes and respective codings, which were identified as major influencing factors in this consensus process. We defined those codes as major influencing factors that both coders (PH, AL) rated as being highly relevant to explain the implementation process in this trial. Reasons for high relevance could be high frequency of codings in process interviews, field notes, and minutes and/or the perceived impact of the influencing factor by the coders. The entire coding scheme including all CFIR constructs and all additional inductive codes is described in Additional File 3.

As a quantitative analysis step, for each code the number of codings in total, the number of coded documents, and the number and percentage of codings in which the influencing factor was classified as a barrier or a facilitator to SDM implementation (cp. meta-codes) were also included in Additional File 3. To avoid over-interpretation of this quantitative step, we translated the ratio between passages classified as barriers and facilitators as follows: “Rather facilitator” if at least 70% of codings were classified as facilitator; “balanced” if less than 70% of codings were classified as facilitator and less than 70% were classified as barrier; and “rather barrier” if at least 70% of codings were classified as barrier.

For qualitative content analysis, we used MAXQDA software (VERBI GmbH, Berlin, Germany). Descriptive statistics of demographic data were conducted with IBM SPSS Statistics 25 (IBM Corp, Armonk, NY).

### Researchers’ characteristics

All researchers involved in this study had comprehensive experiences in conducting qualitative interviews and qualitative content analysis prior to this trial. Process interviews were conducted by WF and JZ. WF is a female health scientist (M.Sc.) and doctoral researcher with experience in oncological health services research. JZ is a female clinical psychologist (Dipl.-Psych.), post-doctoral researcher, and licensed psychotherapist working with cancer patients. Both had prior experience in collecting qualitative data. PH and AL analyzed the process evaluation data. PH is a female clinical psychologist (Dipl.-Psych.), post-doctoral researcher, licensed psychotherapist and trained psycho-oncologist. AL is a female neurocognitive psychologist (M.Sc.), doctoral researcher, and trained psycho-oncologist. PH and AL had both conducted qualitative research before and were familiar with qualitative content analysis. IS supervised the trial as its principal investigator. She is a female clinical psychologist (Dipl.-Psych.), senior researcher, licensed psychotherapist, and trained psycho-oncologist with comprehensive knowledge and experience in qualitative research and implementation research.

## Results

### Description of data sets and sample characteristics

We asked 164 eligible HCPs to take part in process interviews. 38 HCPs refused to take part in interviews due to the following reasons: no time (*n* = 27, 71.1%), had not heard about the study (*n* = 3, 7.9%), feeling sick (*n* = 2, 5.3%), no interest in participation (*n* = 1, 2.6%), already participated during the current implementation interval (*n* = 1, 2.6%), or for unknown reasons (*n* = 4, 10.5%). Participation in multiple interviews was possible in different implementation phases, but not within one phase. 126 HCPs agreed to participate. Ten interviews were performed as a group interview with up to four interviewees. Thus, 107 interview transcripts were available for analysis. For characteristics of the process interviews including number of interviews, number of interview participants and duration of interviews, see Table 1.

**Table 1:** Characteristics of the data sets

		***n***	***%***
**Process interviews**	**Total number of interviews**	107	100.0
	**Participants per interview**		
	Interviews with 1 interviewee	92	86.0
	Interviews with 2 interviewees	13	12.1
	Interviews with 4 interviewees	2	1.9
	**Interviews per department**		
	Department 1	61	57.0
	Department 2	25	23.4
	Department 3	21	19.6
	**Interviews per clinical setting**		
	Inpatient wards	81	75.7
	Outpatient clinics	26	24.3
		***mean***	***range***
	Duration of interviews in minutes	7.8	1.3 – 27.5
		***n (documents)***	***n (pages)***
**Field notes**	**Total number of field notes**	66	304
	Preparation phase	4	11
	Implementation intervals	24	195
	Evaluation waves	38	98
**Meeting minutes**	**Total number of meeting minutes**	39	125
	SDM group training	13	55
	Meeting about quality management documents	1	1
	MDTM reflection meetings with clinical stakeholders	5	33
	Other meetings with clinical cooperation partner	19	36

^a^Participation in multiple interviews was possible in different implementation phases, but not within one phase

Most interviewees were younger than 30 years (47.6%), female (68.3%), nurses (54.0%), and had a working experience of less than 5 years (60.3%). For details on demographic data of interview participants, see Table 2.

**Table 2:** Demographic data of participants of process interviews

		***n***	***%***
**Total number of participants**		126	100.0
**Age **^**a**^	< 30 years	60	47.6
	31–40 years	37	29.4
	41–50 years	14	11.1
	> 50 years	12	9.5
	Missing	3	2.4
**Gender**	Female	86	68.3
	Male	40	31.7
**Position**	Nurse	68	54.0
	Nurse in leadership position	4	3.2
	Medical assistant ^b^	5	4.0
	Junior physician	39	30.9
	Senior physician	7	5.6
	Others	3	2.4
**Work experience in oncology**	< 5 years	76	60.3
	5–10 years	23	18.3
	11–20 years	15	11.9
	> 20 years	11	8.7
	Missing	1	0.8

^a^Age was assessed in categories of about 10 years each within the working age range to allow description of the sample without compromizing anonymity; ^b^ Medical assistant = German position of “Medizinische Fachangestellte”

Field note documentation comprised a total of 304 pages in four documents collected during the preparation phase of the trial, 24 documents collected during the three implementation intervals, and 38 documents collected during the four evaluation waves. Additionally, 125 pages in 39 documents with meeting minutes comprised minutes of the following occasions: 13 SDM group training sessions, five MDTM reflection meetings with clinical stakeholders, one meeting on quality management documents, and 19 meetings with clinical cooperation partners (e.g., meetings with nurses in leadership positions to discuss the distribution of Ask3Q and information materials on their ward). For detailed characteristics of field notes and meeting minutes, see Table 1.

### Influencing factors on SDM implementation

Of the 79 potential influencing factors in the entire coding scheme, we deductively defined 53 (67.1%) and inductively added 26 (32.9%). From these 79 factors, we identified a total of 28 major influencing factors (35.4%). Half were deductively and half were inductively derived. Eighteen of the deductively defined influencing factors from the CFIR were not found at all.

**Fig. 2:**
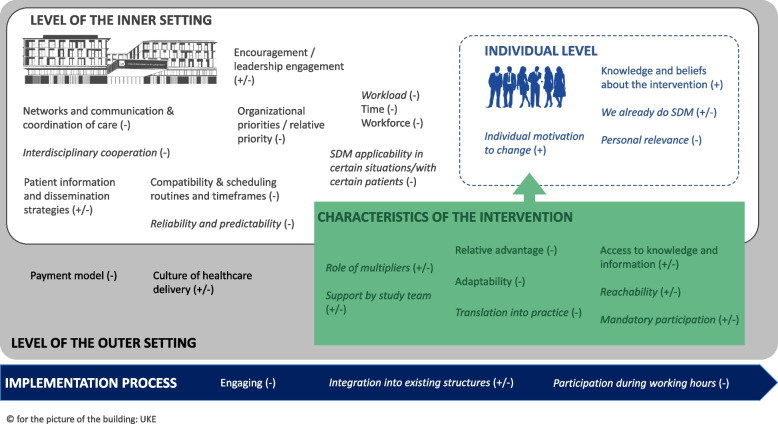
Major influencing factors in this SDM implementation trial Notes: *italics* indicate inductively derived influencing factors; ( +) indicates that the factor was rather a facilitator for SDM implementation, ( ±) indicates that the factor was balanced between facilitator and barrier, (-) indicates that the factor was rather a barrier for SDM implementation

In the following paragraphs, we present the results of our qualitative content analysis for major influencing factors on SDM implementation in this trial (see Fig. 2). Names of codes are written in italics for easier recognition and all inductively derived influencing factors were labeled accordingly. The complete coding scheme including all deductive and inductive codes with theoretically deduced definitions, descriptions and quotes from the empirical codings, and some quantitative results can be found in Additional File 3.

### Major influencing factors regarding characteristics of the individuals involved

Regarding *Knowledge and beliefs about the intervention*, some of the interviewed HCPs reported no or very little knowledge of SDM and the implementation program. Participants, who knew about it, voiced predominantly positive attitudes towards SDM and the implementation program. At the same time, participants did not see many possibilities to implement SDM and the implementation strategies at their department. Reasons were assumed patient preferences and concerns that SDM would require more time and lead to longer clinical encounters.

*“I have also discussed this [implementation of SDM in the department] in the multiplier training and I still find it totally difficult, so I think the topic is totally important and it's great that the project exists, but this implementation path, well, it's just really difficult.” (Interview I043, dept. 1, nurse)*

If asked about their attitudes towards the Ask3Q implementation strategy, it was mostly perceived as helpful. Some HCPs had seen the Ask3Q posters and postcards, but did not realize the connection to SDM or to our implementation program. Attitudes towards SDM group training were mostly positive, but some nurses described the training as too theoretical and scientific. Coaching was perceived as helpful and coaching feedback was well adopted. Some physicians doubted the benefit of SDM implementation in MDTMs. Overall, we classified the knowledge and beliefs about SDM reported in this study as rather a facilitator due to the predominantly positive attitudes. As subordinate major influencing factors, we identified *We already do SDM* and a lack of *Personal relevance* (both inductively derived). The belief of some HCPs to already perform SDM was classified as balanced between barrier and facilitator. Some HCPs stated that they already motivate patients to ask questions and to read patient information material. Furthermore, some participants reported that some contents of our SDM training were already known to them and patient preferences were already sufficiently integrated in MDTMs. Those attitudes might have influenced the perceived personal relevance of the trial.

*“I'm a bit older now and do it [SDM] that way anyway. Where I think that the patient is not at all receptive to it, perhaps less so.” (Interview I079, dept. 2, physician)*

A lack of perceived personal relevance was classified as a barrier to SDM implementation. Sometimes nurses did not perceive it as their responsibility to perform SDM. Supporting patients in decision-making and implementing SDM in the department was perceived as a physicians’ task.

*“As a nurse, I have not yet dealt with this in such an extreme way, because it primarily concerns the physicians, because the physicians have to show the patients all these paths. And - and nurses can't really do that much.” (Interview I069, dept. 2, nurse)*

Additionally, some junior physicians explained that they were not discussing treatment decisions with patients, but this was the task of senior physicians.

In addition, *Individual motivation to change* was inductively found to vary greatly between individuals, which might have influenced participation in the SDM implementation program. People, who were not interested in SDM or believed to already perform SDM were perceived to be less motivated to take part in the implementation strategies. However, this influencing factor was classified as rather a facilitator in this trial, suggesting that the majority of HCPs in this study voiced a high motivation to change towards more SDM.

### Major influencing factors regarding the inner setting

Most major influencing factors were found on the organizational level (corresponding to the inner setting in CFIR), with a total of eleven major influencing factors.

Data showed that *Encouragement and leadership engagement* of stakeholders in leading positions (e.g., senior physicians and leading nurses) differed a lot between individuals. Some clinical leaders supported the research team in organizing the implementation and motivating their colleagues to take part in the implementation program. Others remained rather passive and did not engage in the implementation process. We found SDM implementation in our trial to be facilitated if clinical leaders talked positively about SDM implementation and our implementation program, drove the implementation of our implementation strategies, motivated their colleagues for participation, and announced SDM group training as mandatory.

*“[A senior physician] also makes it clear that it would be very helpful if [the chief physician] would send an e-mail stating that all physicians are obligated to participate in the team training. Here, it would be necessary to involve the management even more and to exert pressure from above.” (Meeting minute, dept. 1)*

Junior physicians in process interviews highlighted the importance of senior physicians as role models for SDM uptake. From their point of view, senior physicians should exempt junior physicians from their service duties to take part in SDM training and compliment junior physicians for performing SDM. Due to the variation between individuals, this influencing factor was classified as balanced between barrier and facilitator for SDM implementation in this trial.

Regarding *Networks, communication, and coordination of care*, team communication was frequently described as poor, mostly due to frequent staff rotations in many divisions of the participating departments. Some nurses in process interviews perceived that patients voiced different issues with physicians and nurses. They reported that patients were more likely to voice questions and concerns to nurses than to physicians.

*"Often questions are more likely to be directed to nurses, in part because patients are not well educated and nurses have much closer contact with patients than physicians." (Field note, dept. 2)*

Furthermore, nurses criticized that pre-treatment consultations on wards often took place during ward rounds, which often did not involve nurses, and that patients were sometimes not aware of all treatment consequences. As a subordinate influencing factor to *Networks, communication, and coordination of care*, a lack of *Interdisciplinary cooperation* was found as an important barrier for SDM implementation by inductive coding. In most inpatient wards and outpatient clinics, no official structures for oral information exchange between nurses and physicians were reported. Accordingly, nurses often did not feel involved in decision-making and wished for joint pre-treatment consultations and interdisciplinary SDM training. Both the superordinate influencing factor *Networks, communication, and coordination of care* and the sub-factor *Interdisciplinary cooperation* were classified as rather being barriers to SDM implementation within this trial.

Regarding organizational resources, *Workload* (inductive), *Time*, and *Workforce* were identified as major influencing factors acting as barriers to SDM implementation. Data showed that limited resources, i.e., lack of time, high workload, and a limited workforce, resulted in clinical consultations where decisions had to be made quickly. As a consequence, it was reported that decision-making was often guided mainly by physicians and patients had less time to think about decisions.

*“One of the [training participants] states that the length and comprehensiveness of the decision-making talks depends very much on the appointments he has afterwards. He notes that he is often unable to conduct such discussions as he would like due to other appointments.” (Meeting minute, dept. 1)*

Lack of time was also very often mentioned as a reason for not taking part in SDM group training or coaching sessions. A strong connection between the different influencing factors regarding organizational resources was found. Participants of process interviews felt that limited resources illustrated the *Organizational priorities and relative priority* on economic and research interests rather than patients’ needs. Additionally, HCPs assumed that patients had other priorities than implementation strategies like Ask3Q during their stay at the hospital. Overall, the organizational priorities and relative priority of SDM were categorized as rather a barrier to SDM implementation in this trial.

In terms of *Patient information dissemination strategies*, inpatient wards and outpatient clinics were reported to differ in their ways of disseminating patient information material, especially the Ask3Q posters and postcards. Hence, this influencing factor was classified as balanced between SDM facilitator and barrier in this trial. In general, most HCPs reported a positive attitude towards Ask3Q and HCPs supported the idea to use it in routine care. On some wards, the Ask3Q postcards were added to routine workflows and disseminated to patients on a regular basis. Most often, nurses were responsible for the dissemination of postcards and other patient information material. However, they wished for physicians to become more involved in this task. It was mentioned to be most effective if postcards were presented in the patients’ rooms and actively handed over to patients, ideally when patients have their first appointment at the outpatient clinic.

*“The first contact is in the outpatient clinic. So it makes most sense, it has to be said, in our outpatient clinic here, that the patients are given the [Ask 3 Questions] perhaps right from the start. So rather - that has to be improved in the outpatient clinic.” (Interview I068, dept. 2, physician)*

SDM implementation was facilitated if implementation strategies were integrated in clinical workflows and routines and timeframes were scheduled accordingly (i.e., *Compatibility, scheduling routines and timeframes*). However, the integration of SDM training in clinical routines was found to be challenging. For example, training sessions would have had to be scheduled months in advance, in order to be able to plan for HCPs to participate during working hours. In reality, participation often happened after the shift.

*“Participation in the multiplier training will generally also be difficult at other times due to very limited time resources. The implementation of the team training is also difficult, as only a maximum time window of 45 minutes will be possible for this; in the normal working day, a longer training is not feasible - not even for the nursing staff alone.” (Meeting minute, dept. 1)*

*Reliability and predictability* of clinical stakeholders, meetings with cooperation partners, and coaching sessions with physicians was inductively detected as a subordinate influencing factor to compatibility, scheduling routines and timeframes. Often, individual coaching sessions of physicians had to be spontaneously arranged or were postponed by physicians due to clinical workflows or limited resources.

*"It's - it's just - you always don't know where you're assigned until the next day and then to remember to let you know, that just makes it a little bit difficult then." (Interview I080, dept. 2, physician)*

It was often not possible to select clinical encounters, where treatment decisions were made, for coaching sessions. Thus, in some coaching sessions, we could only provide feedback on communication in general due to a lack of SDM relevant content. Furthermore, some stakeholders who signed up for SDM training, coaching, or other meetings did not appear or did not answer to e-mails from the project team.

Both the difficulties regarding compatibility, scheduling routines, and timeframes and reliability and predictability were found to rather be barriers in this trial.

Regarding patients’ needs and resources, a further important influencing factor was *SDM applicability in certain situations and with certain patients* (inductively coded). For example, some HCPs perceived that SDM was less applicable for specific cancer entities like leukemia or in emergency situations. HCPs also reported that SDM cannot be applied with patients in specific clinical conditions, for example with patients who were overwhelmed by their diagnosis, were rather passive, or had limited cognitive capacities. Additionally, SDM was perceived not to fit with the clinical pathways for certain patients and/or situations. This influencing factor is interrelated with HCPs’ individual beliefs about SDM mentioned above.

### Major influencing factors regarding the outer setting

We found two major influencing factors on the level of the health care system (corresponding to the outer setting in CFIR): *Payment models* and the *Culture of health care delivery*.

Regarding *Payment models*, participants described that the German health care system does not reimburse explaining and discussing treatment options with patients. Furthermore, the payment amount was reported to differ between treatments. Our data suggested that physicians sometimes did not discuss specific treatment options with their patients, because they were less rentable. We classified the influencing direction of this factor as rather a barrier to SDM implementation, and found a strong connection to two other major influencing factors, *Culture of health care delivery* and *Organizational priorities and relative priority*.

*“Well, I mean - that always sounds so harsh, doesn't it? But at the end of the day, of course, you're a business enterprise at the end of the day, and - well, I very rarely hear a physician say, okay, I'd think about that if I were you.” (Interview I045, dept. 3, nurse)*

By some interviewees, the current *Culture of health care delivery* in Germany was perceived to contradict SDM. Some participants argued that the current culture of our health care system called for patients being offered and receiving maximum care. This might lead to surgeries being performed at high cost and other options like active surveillance or best supportive care being less prominent. However, only about two thirds of codings within this influencing factor were classified as barriers to SDM, in about a third of coded passages the direction of influence of the culture of health care delivery was classified as a facilitator. Codings in which the culture of health care delivery was perceived as facilitating SDM were for example related to the introduction of a patient activation strategy initiated by the city of Hamburg. Thus, culture of health care delivery seemed to be a major influencing factor, but was perceived either as a facilitator or barrier to SDM implementation by different participants.

### Major influencing factors regarding characteristics of the intervention

Eight major influencing factors concerning attributes of the intervention and the implementation strategies were identified.

After the multiplier training sessions, some participating physicians and nurses voiced problems with their role. They reported to feel insecure with offering SDM team training sessions to their colleagues with the help of the research team.

*“And then it was also said that we should actually do the [team] training and I found that a bit difficult, because you learned that on this day, but you are not directly a professional in this area.” (Interview I062, dept. 2, nurse)*

As a consequence, they often did not take an active part in subsequent SDM team training for their colleagues. The research team often drove large parts of the team training or executed them without multipliers. This was summarized by inductive coding in the two major influencing factors *Role of multipliers* and *Support by study team*. Both codes were closely connected and classified as balanced between barrier and facilitator.

If HCPs perceived a clear *Relative advantage* of SDM for their own daily work, participation in the SDM implementation program could be facilitated. However, within this process evaluation, we rather found a lack of perceived relative advantage of SDM, thus this factor had to be classified as rather a barrier. Interviewees called for being made aware of potential advantages participation offered in order to be more motivated to participate in the implementation program.

*“From the beginning, there was a bit of a problem (…), that the physicians understand from the outset what they can extract in terms of positive effects for the efficiency of everyday life, and that was a bit lacking. [... This study was] so to speak primarily perceived only as additional work [...].” (Interview I094, dept. 3, physician)*

Furthermore, *Adaptability* of some implementation strategies of the program was necessary to make their execution possible. For example, some wards decided against using the Ask3Q posters, but for the postcards. Also, timing and duration of SDM training were adapted to meet capacities of participants. However, in this trial adaptability was classified as rather a barrier to successful SDM implementation.

An easy *Translation into practice* of SDM and the SDM implementation strategies was found to be crucial for implementation (inductive code). According to our data, some HCPs perceived transfer of SDM into practice as difficult and had the impression that the concept of SDM is too theoretical for straightforward implementation into practice. Hence, translation into practice was rather a barrier in this trial.

*“I found the [SDM training] quite interesting, but I think for many of my colleagues and I it is very - in my opinion - a very theoretical concept, which I think is often not practically feasible. The idea is - a lot of it was correct and interesting - but I don't think it can be implemented one-to-one with our patients.” (Interview I024, dept. 1, physician)*

Also, the perception of *Access to knowledge and information* about SDM and the implementation program differed between HCPs. The project team used various pathways to inform HCPs about the implementation program: e.g., information provision by clinical cooperation partners such as leading nurses of respective wards, handouts or training manuals sent by mail, and information on the project’s progress via e-mail or in regular meetings. Nevertheless, some HCPs reported that they had not received information about the implementation program at all or not in time. In addition, data showed that *Reachability* of HCPs differed between professions (inductive code). Nurses preferred to get information from individual mailboxes (most preferred option), nurses in leadership positions, the regular hospital-wide newsletters, or personal approach by the study team. Sending information to nurses by email would not have been beneficial, since almost all nurses stated to not read e-mails regularly or at all. In contrast, physicians preferred to receive information via e-mail or during regular meetings. Additionally, participants suggested to announce *Mandatory participation* for SDM training and coaching sessions to increase participation numbers (inductive code).

*“When implementing the Ask 3 Questions, we would have to consider how important it is that patients are given the cards directly. If this was presented to the physicians as an option, it would not be implemented. This would have to be made obligatory if it were to actually happen.” (Field note, dep. 3)*

The superordinate influencing factor *Access to knowledge and information* and its sub-factors *Reachability* and *Mandatory participation* were classified as balanced between barrier and facilitator.

### Major influencing factors regarding the implementation process

Concerning the process factor *Engaging*, data showed that clinical cooperation partners of the trial had generally been very supportive. Nevertheless, it also occurred that cooperation partners did not show up to scheduled meetings or were not reachable by e-mail or phone. Since those instances were perceived as impeding the implementation process, engaging was classified as rather a barrier.

Regarding the execution of the trial, it was perceived as a barrier, if HCPs had to participate in SDM group training in their off time, especially directly after a shift.

*“By chance, I also met [a physician cooperation partner], brief conversation about the trainings. Many of the physicians directly approached said that 5-hour training sessions were too much after work and that many were happy to finally call it a day. Most of them liked the cooperation as long as it was within their working hours.” (Field note, dep. 1)*

Difficulties in meeting the need for *Participation during working hours* was classified inductively as a major influencing factor that rather posed a barrier to SDM implementation. On the other hand, if training sessions were to be scheduled during working hours, training duration would have to be shortened due to limited time resources. In addition, *Integration into existing structures* was inductively found to be a major influencing factor. Especially SDM training was called to be integrated into existing structures.

*“I think that's the problem with the appointments. I think the advantage is, of course, if you now have fixed appointments where everyone goes, [a regular internal training] or so, that then, I think, the participation would be somewhat higher, as if it is in the clinic everyday life. Then always something comes in between and then that is rather an appointment where you say, you skip it.” (Interview I056, dept. 1, physician)*

Integration into existing structures was classified as balanced between barrier and facilitator, since we frequently managed to embed training sessions into regular meetings, but sometimes encountered difficulties to do so. Whether participation was possible during working hours and integrated into existing structures or not was found to be closely connected to the influence of compatibility, scheduling routines, and timeframes on the organizational level.

## Discussion

We performed a comprehensive qualitative process evaluation of a theoretically and empirically based SDM implementation program with six strategies within a cluster-randomized SDM implementation trial. Data collection integrated various stakeholders’ perspectives through qualitative process interviews with nurses and physicians of participating departments, field notes by the study team, and meeting minutes. Qualitative data analysis was based on the CFIR, a conceptual framework for implementation research. Major influencing factors on SDM implementation were found for all domains of the CFIR and amended by inductively derived influencing factors where necessary. Major themes regarded e.g., relative priority and relevance of SDM and its implementation, leadership support, coordination and communication, applicability and compatibility, and available resources. In addition, strong interrelations between different major influencing factors were identified.

The influencing factors found in this trial align with previously reported influencing factors on SDM implementation [10, 11, 14–17]. For example, the question whether SDM is applicable in certain clinical situations or for patients with certain characteristics has been previously found as one of the most often named barriers to SDM in a Cochrane review [14]. In addition, especially the influencing factors regarding the inner setting highlighted the importance to pay attention to hierarchical structures within teams for SDM implementation. Further, our results replicated that finances and payment models seem to play a major role for the lack of SDM implementation [16, 39]. On one hand, SDM uptake was found to be limited by lack of organizational resources to perform SDM. This aligns with prior publications suggesting that reimbursement for SDM uptake might foster SDM implementation [16, 39]. Reflecting on available resources beforehand, as suggested by the National Cancer Institute [40], and if possible, arranging for additional resources could help to avoid limited SDM implementation success due to lack of resources. On the other hand, some treatment options were reported to be more lucrative than others. Participants in this trial perceived that the differences in reimbursement for different treatment options might lead to a bias in the presentation of different treatment options to patients. If this was the case, it would also limit SDM. This is in line with an interview study with cancer care stakeholders in the United States of America, which found that financial interests of healthcare organizations function as barriers to SDM implementation [39].

Furthermore, our comprehensive assessment of influencing factors guided by the CFIR as a theoretical foundation added to existing literature by highlighting the interrelations between influencing factors on different levels (i.e. regarding different CFIR domains) that likely need to be addressed simultaneously. A successful implementation program might need to take into account many if not all of the influencing factors in order to lead to a relevant change in SDM implementation. Barriers on different levels might stabilize the current lack of SDM uptake. Implementation efforts that manage to tackle some, but not enough of the influencing factors are likely not finding significant effects. This might explain, why the outcome evaluation of this trial did not show significant differences in patient-reported SDM uptake and most secondary outcomes before and after implementation of the multi-component SDM implementation program [20]. In the quantitative process evaluation of the trial, low reach and adaptations in dose were discussed as possible explanations for the lack of effects [20]. These might in turn be explained by the influencing factors found in the qualitative process evaluation at hand. For example, a lack of resources, compatibility, and personal relevance combined with organizational priorities lying elsewhere might have led to limited reach, adaptations in dose, and consequently lack of effects on SDM implementation.

In addition, the influencing factors differ in who has the power to modify them and how. Positive attitudes and motivation to change on the individual level, as found in this trial, did not suffice to foster SDM uptake on the department level. This aligns with recent literature that points to the necessity to consider barriers on the organizational and health system level in order to successfully implement SDM [16, 39]. However, we might need different strategies to have an effect on the organization, the health system, and individual characteristics. Organizational influencing factors such as leadership engagement, relative priority, and interdisciplinary communication might be changeable on the department or hospital level. Payment models or organizational resources such as time or workforce might not even be in the hands of individual organizations, but be decided on an even higher policy level. As a recent development in Germany, the Federal Joint Committee (German: Gemeinsamer Bundesausschuss) recommended the transfer of SDM into routine health care in February 2023 [41]. This has the potential to facilitate increased policy support for SDM in Germany – including potential reimbursement. In order to achieve the necessary structural changes and change the culture of routine health care delivery, influencing factors on all levels including the outer setting need to be evaluated using appropriate tools [42]. Nevertheless, some influencing factors might have to be defined as unmodifiable [43].

Participatory research could be a way out of limited implementation success due to influencing factors on the individual, organizational, and intervention level [44, 45]. Although we followed several participatory research approaches such as maintaining close partnership with clinical cooperators, promoting participation, or supportive monitoring by the study team [45], a stronger emphasis on co-design could have benefitted this trial. A recent implementation study on SDM in breast cancer care that used a co-design approach found effects in observer-assessed SDM uptake, but also no effects in patient-reported SDM [21]. In addition, another multi-component implementation trial from Germany has a strong focus on developing disease-specific patient decision aids together with clinical partners also reported and was able to temporarily allocate clinical departments’ work force resources to the development of these [19, 46]. This trial found significant effects on SDM uptake in preliminary results from one department and reported a reach of over 90% [46].

### Strengths and limitations

This multi-faceted implementation trial can guide future research with an exemplary function. We followed established frameworks and recommendations for implementation research [25, 47], planned a priori, and continuously conducted a comprehensive process evaluation, and thoroughly executed the study protocol [22]. The CFIR [25] as a theoretical basis guiding the process from planning to analysis and interpretation of results is a major strength. In addition, the trial has high ecological validity. For interviews, we used a purposive sampling approach aiming for maximum variation in HCPs who worked on inpatient wards and at outpatient clinics of three departments of a German comprehensive cancer center. The vast majority of interviews was conducted on the spot at the ward or clinic. Interviewers were not involved in execution of the implementation program or outcome evaluation of the trial. The interviews were further complemented by field notes by the study team (i.e., from an outside perspective) and meeting minutes. Furthermore, the thorough data analysis involved a comprehensive quality control conducted by the two coders.

However, generalizability has to be assumed with caution since this was a single-center study including three departments only. Replication of the findings in other comprehensive cancer centers and countries is necessary. Furthermore, the sampling procedure could limit fair representation of the target population. Potential interview participants were contacted on the corridors of the inpatient wards and outpatient clinics. Nurses in leadership positions and senior physicians could be underrepresented, because they more often work in their offices compared to ward nurses or junior physicians. In terms of data saturation, a more targeted recruitment strategy might have been useful to ensure completeness. Additionally, it was not possible to conduct minutes for all meetings with clinical cooperation partners and SDM team trainings due to personnel resources. Nevertheless, we were able to collect rich data sets and identify influencing factors regarding all CFIR domains. The extensive coding system with a total of 79 codes and strong interdependency between individual codes made data analysis challenging. Quality of data analysis was safeguarded by extensive peer-review within the study team. Nevertheless, reducing the findings of this study to a sparser framework and corroborating the findings of this study with a quantitative design in the future would be valuable next steps. Furthermore, an in-depth analysis of potentially diverging views across professions and profession-specific needs could further enrich our understanding.

## Conclusion

The comprehensive process evaluation at hand was theoretically based on the CFIR, complements the outcome evaluation of the SDM implementation trial, and adds to our understanding of influencing factors on SDM implementation. To our knowledge this is the first SDM implementation trial following a stepped-wedge cluster randomized design including such a thorough a priori planned process evaluation. Major influencing factors were found for all domains of the CFIR, and strong interrelations between those factors were evident. The identified influencing factors can be used for planning, conducting, and evaluating future SDM implementation. Further studies should investigate which of the influencing factors are predictors for implementation success, and how the interrelations can be taken into account successfully. This knowledge, in turn, may support the implementation of SDM in routine care in the future.

### Supplementary Information

**Additional file 1: **COREQ (COnsolidated criteria for REporting Qualitative research) Checklist.

**Additional file 2: **Interview guide for process interviews with health care professionals

**Additional file 3: **Description of all codes, qualitative codings, quotes, and quantitative analysis of the direction of the influence.

## Data Availability

Deidentified data that support the findings of this study are available on reasonable request. Investigators who propose to use the data have to provide a methodologically sound proposal directed to the corresponding author. Signing a data use/sharing agreement will be necessary, and data security regulations both in Germany and in the country of the investigator who proposes to use the data must be complied with. Due to the data being closely connected with the participating departments, it might not be possible to sufficiently anonymize all persons in all documents within this data set. Thus, some documents might have to be excluded from sharing. Preparing the data set for use by other investigators requires substantial work and is thus linked to available or provided resources. The data sets used in this study are in German language.

## Abbreviations

AL: Anja Lindig
Ask3Q: Ask 3 Questions (patient activation strategy)
CFIR: Consolidated Framework for Implementation Research
COREQ: Consolidated criteria for reporting qualitative research checklist
HC: Hannah Cords (cp. acknowledgements)
HCP: Health care professional
HH: Henning Hanken
IS: Isabelle Scholl
JZ: Jördis Zill
MDTM: Multidisciplinary team meeting
MP: Mia-Carlotta Peters
PH: Pola Hahlweg
PREPARED: Acronym of the trial “Evaluation of a program for routine implementation of shared decision-making in cancer care: a stepped wedge cluster randomized trial”
SDM: Shared decision-making
UCCH: University Cancer Center Hamburg
UKE: University Medical Center Hamburg-Eppendorf
VM: Volkmar Müller
WF: Wiebke Frerichs
