# Supplementary material for: Major influencing factors on routine implementation of shared decision-making in cancer care: qualitative process evaluation of a stepped-wedge cluster randomized trial
Source: BMC Health Serv Res. 2023 Aug 8;23:840. doi: 10.1186/s12913-023-09778-w (PMC10408234; doi:10.1186/s12913-023-09778-w)
Supplement: Supplementary file 1 — Additional file 1: COREQ (COnsolidated criteria for REporting Qualitative research) Checklist. [file 12913_2023_9778_MOESM1_ESM.pdf]

Additional file 1: COREQ (COnsolidated criteria for REporting Qualitative research) Checklist

Note: The page numbers in this document refer to the clean copy of the "R2" revised version of the manuscript.

**Topic**	**Item No**	**Guide Questions/Description**	**Reported on page No.**
**Domain 1: Research team and reflexivity**			
Personal characteristics			
Interviewer/facilitator	1	Which author/s conducted the interview or focus group?	See page 8 and 9, section "Researcher characteristics"
Credentials	2	What were the researcher's credentials? E.g. PhD, MD	See page 8 and 9, section "Researcher characteristics"
Occupation	3	What was their occupation at the time of the study?	See page 8 and 9, section "Researcher characteristics""
Gender	4	Was the researcher male or female?	See page 8 and 9, section "Researcher characteristics"
Experience and training	5	What experience or training did the researcher have?	See page 8 and 9, section "Researcher characteristics"
Relationship with participants			
Relationship with participants	6	Was a relationship established prior to study commencement?	See page 6, section "Data collection"
Participant knowledge of the interviewer	7	What did the participants know about the researcher? e.g. personal goals, reasons for doing the research	See page 6, section "Data collection"
Interviewer characteristics	8	What characteristics were reported about the interviewer/facilitator? e.g. Bias, assumptions, reasons and interests in the research topic	See page 7 and 8, section "Researcher characteristics"
**Domain 2: Study design**			
Theoretical framework			
Methodological orientation and Theory	9	What methodological orientation was stated to underpin the study? e.g. grounded theory, discourse analysis, ethnography, phenomenology, content analysis	See page 7 and 8, section "Data analysis"
Participant selection			
Sampling	10	How were participants selected? e.g. purposive, convenience, consecutive, snowball	See page 5 and 6, section "Data collection"

Method of approach	11	How were participants approached? e.g. face-to-face, telephone, mail, email	See page 6, section “Data collection”
Sample size	12	How many participants were in the study?	See page 9, section “Description of data sets and sample characteristics”
Non-participation	13	How many people refused to participate or dropped out? Reasons?	See page 9, section “Description of data sets and sample characteristics
Setting			
Setting of data collection	14	Where was the data collected? e.g. home, clinic, workplace	See page 5 and 6, section “Data collection”
Presence of non-participants	15	Was anyone else present besides the participants and researchers?	See page 6, section “Data collection”
Description of sample	16	What are the important characteristics of the sample? e.g. demographic data, date	See page 9, section “Description of data sets and sample characteristics” and Tables 1 and 2
Data collection			
Interview guide	17	Were questions, prompts, guides provided by the authors? Was it pilot tested?	See page 6, section “Data collection” and Additional File 2
Repeat interviews	18	Were repeat interviews carried out? If yes, how many?	See page 5 and 6, section “Data collection”
Audio / visual recording	19	Did the research use audio or visual recording to collect the data?	See page 6, section “Data collection”
Field notes	20	Were field notes made during and/or after the interview or focus group?	See page 6, section “Data collection”
Duration	21	What was the duration of the interviews or focus group?	See page 9, section “Description of data sets and sample characteristics”
Data saturation	22	Was data saturation discussed?	See page 6, section “Data collection”
Transcripts returned	23	Were transcripts returned to participants for comment and/or correction?	See page 6, section “Data collection”
**Domain 3: analysis and findings**			
Data analysis			

Number of data coders	24	How many data coders coded the data?	See page 7 and 8, section “Data analysis”
Description of the coding tree	25	Did authors provide a description of the coding tree?	See Additional File 3
Derivation of themes	26	Were themes identified in advance or derived from the data?	See page 7 and 8, section “Data analysis”
Software	27	What software, if applicable, was used to manage the data?	See page 8, section “Data analysis”
Participant checking	28	Did participants provide feedback on the findings?	See page 6, section “Data collection”
Reporting			
Quotations presented	29	Were participant quotations presented to illustrate the themes/findings? Was each quotation identified? e.g. participant number	See page 10 to 19 (results section) and Additional file 3
Data and findings consistent	30	Was there consistency between the data presented and the findings?	See page 10 to 19 (results section)
Clarity of major themes	31	Were major themes clearly presented in the findings?	See page 10 to 19 (results section) and Additional file 3
Clarity of minor themes	32	Is there a description of diverse cases or discussion of minor themes?	See page 10 to 19 (results section) and 19 to 21 (discussion section)

Tong A, Sainsbury P, Craig J. Consolidated criteria for reporting qualitative research (COREQ): a 32-item checklist for interviews and focus groups. International Journal for Quality in Health Care. 2007. Volume 19, Number 6: pp. 349 – 357
