# Supplementary material for: Major influencing factors on routine implementation of shared decision-making in cancer care: qualitative process evaluation of a stepped-wedge cluster randomized trial
Source: BMC Health Serv Res. 2023 Aug 8;23:840. doi: 10.1186/s12913-023-09778-w (PMC10408234; doi:10.1186/s12913-023-09778-w)
Supplement: Supplementary file 2 — Additional file 2: Interview guide for process interviews with health care professionals [file 12913_2023_9778_MOESM2_ESM.pdf]

**Additional File 2: Interview guide for process interviews with health care professionals**

<table>
<tr><td>1.</td><td>Introduction of the interviewer and interviewee recruitment<ul><li>Short introduction of the interviewer and the implementation program and trial</li><li>Recruitment of the participant for a short interview, if necessary: making a later appointment</li><li>Information on audio-recording, transcription of audio-recordings and process of anonymisation, gathering oral consent for participation</li></ul></td></tr>
<tr><td>2.</td><td>Asking for knowledge about the implementation program and trial<ul><li>“Have you already heard about the implementation program and trial “Behandlungswege gemeinsam entscheiden”? It is also called PREPARED trial.”<br><br>If yes:<ul><li>“What do you know about the PREPARED implementation program and trial?”</li><li>“Who informed you about the PREPARED implementation program and trial?”</li></ul></li></ul></td></tr>
<tr><td>3.</td><td>Asking for knowledge about single strategies of the implementation program<ul><li>“The implementation program aims to facilitate shared decision-making in this department. For shared decision-making, patients and healthcare professionals equally and actively take part in the decision-making process to decide about the further treatment plan. In this study, we used several implementation components like team training on shared decision-making and dissemination of patient information material. Have you ever heard of these implementation strategies and what do you know about them?”</li><li>If necessary, interviewer shows a chart displaying the six strategies of the implementation program and the Ask 3 Questions postcard:<ul><li>“Have you heard about one of these strategies?”</li><li>“Have you ever seen this postcard or the equivalent posters in the inpatient clinic and outpatient wards of this department?</li></ul></li><li>If none of the strategies or the implementation program in general are known at all, the interview will be finished</li></ul></td></tr>
<tr><td>4.</td><td>Asking for reachability of interviewees and communication about the implementation program, its strategies and the trial within the department<ul><li>“How did you get information about the implementation program and trial?”</li><li>“Did you like to get information by e-mail / your colleagues / your supervisor / the study team?”<br><br>If no:<ul><li>“What is your preferred way of receiving information (e.g. face-to-face, e-mail) and when would you like to get information?”</li></ul></li><li>For physicians: “Are you part of the email-distribution list of this department?”</li></ul></td></tr>
<tr><td>5.</td><td>Asking for prior participation in the implementation strategies<br><br>[Comment: This question was tailored depending on what the interviewee had mentioned before.]</td></tr>
</table>

| | |
|---|---|
| | • "You just told me that you have been informed about the PREPARED implementation program and trial and/or single strategies by e-mail / your colleagues / your supervisor / the study team. Did you already take part in one of the six components?"<br><br>If yes:<br>• "How did you experience your participation?"<br>• "What do you think about the team trainings / the individual coaching / dissemination of patient information materials?"<br><br>If no:<br>• "Do you plan to participate in one of the six components?"<br>• "What are reasons for your non-participation?" |
| 6. | **Asking for attitudes towards implementation strategies, which are known by the interviewee**<br><br>*[Comment: The following questions were tailored depending on what the interviewee had mentioned before.]*<br><br>• "You told me about your participation the team trainings / the individual coaching / dissemination of patient information materials. What do you think about these components? Can you imagine to implement these components in your daily work in the department?"<br><br>• "Did your participation in the team trainings / the individual coaching / dissemination of patient information materials helped you to perform shared decision-making?" |
| 7. | **Asking for feedback and general comments**<br><br>• "Are there further topics on shared decision-making or the PREPARED implementation program and trial you want to share with me?" |
| 8. | **Asking for demographic information**<br><br>• "What is your profession / function in this ward?"<br>• "How many years have you been working in an oncological setting so far?"<br>• Interviewer shows a table with four age ranges: "Which age range do you belong to?" *[Comment: Age groups were used to allow a description of the sample without compromising anonymity.]* |
