# Supplementary material for: Major influencing factors on routine implementation of shared decision-making in cancer care: qualitative process evaluation of a stepped-wedge cluster randomized trial
Source: BMC Health Serv Res. 2023 Aug 8;23:840. doi: 10.1186/s12913-023-09778-w (PMC10408234; doi:10.1186/s12913-023-09778-w)
Supplement: Supplementary file 3 — Additional file 3: Description of all codes, qualitative codings, quotes, and quantitative analysis of the direction of the influence. [file 12913_2023_9778_MOESM3_ESM.pdf]

Hahlweg, Lindig et al. Major influencing factors on routine implementation of shared decision-making in cancer care: a qualitative process evaluation of a stepped-wedge cluster randomized trial.

# Additional File 3. Description of all codes, qualitative codings, quotes, and quantitative analysis of the direction of the influence.

**Notes:** Light blue background indicates major influencing factor; IF = influencing factor; CFIR = Consolidated Framework for Implementation Research (Damschroder LJ, Aron DC, Keith RE, Kirsh SR, Alexander JA, Lowery JC. Fostering implementation of health services research findings into practice: A consolidated framework for advancing implementation science. Implement Sci. 2009;4:50.); SR = Scoping review (Scholl I, LaRussa A, Hahlweg P, Kobrin S, Elwyn G. Organizational- and system-level characteristics that influence implementation of shared decision-making and strategies to address them - a scoping review. Implement Sci. 2018;13(1):40.); [a] rather facilitator = at least 70% facilitator, balanced = less than 70% facilitator and less than 70% barrier, rather barrier = at least 70% barrier (due to the nature of the data set, margins were defined with great caution to not over-interpret findings, percentages not adding up to 100 are due to inconclusive direction of some codings); * In CFIR these codes are assigned to the domain “Readiness for Implementation (Inner Setting)”; ** In CFIR these codes are assigned to the domain „Implementation Climate (Inner Setting)”; *** In CFIR these codes are assigned to the dimension “Outer setting”; *** The CFIR domain “External Policy & Incentives” was split up according to the domains of the scoping review.

| | Code | Theoretical description | Source | Relevance rating | Qualitative analysis | | | Quantitative analysis | | | | | | |
|---|---|---|---|---|---|---|---|---|---|---|---|---|---|---|
| | | | | | Summary of codings | Quotes | Memos | Codings | Documents with codings | Facilitator | | Barrier | | |
| | | | | | | | | n | n | n | % | n | % | in words [a] |
| I | **Characteristics of the individuals involved** | | | | | | | | | | | | | |
| I1 | **Knowledge and beliefs about the intervention** | CFIR: “Individuals’ attitudes toward and value placed on the intervention as well as familiarity with facts, truths, and principles related to the intervention.” | CFIR | Major IF | Some participants had only little / no knowledge about the study. | | Codings reflect primarily beliefs about the intervention.<br><br>This code was applied to statements about a specific implementation strategy, the entire implementation program, and SDM in general. | 205 | 113 | 150 | 73,2 | 61 | 29,8 | rather facilitator |
| | | | | | Predominantly positive attitudes toward SDM and the implementation program.<br>- SDM is perceived as important and worthy of support, especially by nurses.<br>- Perceived need for SDM<br>- Study promotes awareness for SDM | "I think it's quite important because there are more and more - there are also more and more patients who want to participate in decision-making, who are also well informed. And also want to be informed. And I think that has increased, so felt are the - it's more about deciding together." (interview I098, dept. 3, physician) | | | | | | | | |
| | | | | | HOWEVER: Limited possibilities are seen for implementation in routine clinical settings.<br>- Nurses seem to be more open to implementation than physicians.<br>- Stated reasons for difficulties implementing SDM regarded applicability in certain situations or with certain patients [cp. O8.2] and lack of time [cp. O4.2].<br>- Wide variation in individuals' level of SDM implementation was described (i.e., some physicians already implemented SDM, others do not want to implement it despite the study). | "I have also discussed this in the multiplier training and I still find it totally difficult, so I think the topic is totally important and it's great that the study exists, but this implementation path, well, it's just really difficult." (interview I043, dept. 1, nurse) | | | | | | | | |
| | | | | | **SDM group training**<br>- Mostly positive feedback on group training<br>- Some nurses perceived content of group training as too theoretical and science-based, and reported difficulties understanding it. [cp. also V6.1]<br>- Some participants found training too long, others appropriate in duration. | "She [the physician] emphasized again and again how important it is to have training in conversation skills, even after 30 years of professional experience. She had also learned something new through our project." (field note, dept. 3) | | | | | | | | |
| | | | | | **Coaching**<br>- Perceived as helpful<br>- Coaching feedback was very well received<br>- Many physicians reflected on feedback and tried to implement some of the suggestions. | "The physician also told me that all colleagues had found the coaching sessions very helpful and had taken a lot away with them. [...]. Overall, she said that these coaching sessions were a great opportunity and that she would like to see more such opportunities for reflection and feedback." (field note, dept. 3) | | | | | | | | |
| | | | | | **Patient activation strategy: Ask3Questions (Ask3Q)**<br>- Generally perceived as helpful<br>- Some participants (especially nurses) voiced difficulties with the intervention (i.e., could not connect the Ask3Questions posters to the PREPARED study, did not understand the purpose of the posters) | "I think it's good that patients and their relatives are made more aware of what they want." (interview I062, dept. 2, nurse) | | | | | | | | |

| | | | | | | | | | | | | | | |
|---|---|---|---|---|---|---|---|---|---|---|---|---|---|---|
| | | | | | **Multidisciplinary team meetings (MDTMs):** Some physicians doubt benefit of implementing SDM in MDTMs. | "[A senior physician] clarifies the benefits of the tumor board from his point of view: The patient is only partially the center of attention. Instead, physicians would learn from each other on a multidisciplinary basis and then decide on the best possible treatment. This increases the quality of treatment, which is in the patient's best interest. The patient's wishes therefore do not belong in the tumor board." (field note, dept. 1) | | | | | | | | |
| I1.1 | We already do SDM | Individuals' perception that SDM is already implemented in their routine care or that specific implementation strategies were already in place prior to the study (e.g. handing out information material, empowering patients to ask questions). | inductive | Major IF | Participants reported that principles of SDM are already being implemented and patients are already being made aware of various options. This was reported to lead to limited perceived need for the SDM implementation program within this study. | "I'm a bit older now and do it [SDM] that way anyway. Where I think that the patient is not at all receptive to it, perhaps less so." (Interview I079, dept. 2, physician) | | 46 | 36 | 19 | 41,3 | 30 | 65,2 | balanced |
| | | | | | **SDM group training:** Some participants perceived that the training contained little new information. | "Participants expressed that they were already implementing many points of SDM ('This [SDM] is our daily bread')" (field note, dept. 2) | | | | | | | | |
| | | | | | **Ask3Q:** Some participants perceived that patients are already animated to ask questions. [cp. O6.1] | "They do a lot of that with us anyway. Our patients ask us lots and lots of questions, and we always ask them to do the same." (interview I045, dept. 1, physician) | | | | | | | | |
| | | | | | **Information materials**: Some participants reported that patients were already made aware of information materials. [cp. O6.1] | "With us, the [patient information] is already predominantly distributed. [...] So - and it is also ordered and pushed by the physicians when things are missing or when there are new materials that we don't know about. That's why I think it's going quite well here." (interview I095, dept. 3, nurse) | | | | | | | | |
| | | | | | **MDTMs**: Some participants reported that patient preferences were already integrated in MDTMs. | "Discussing tumor board [= MDTM] results using SDM is a given, he said." (field note, dept. 1) | | | | | | | | |
| I1.2 | Personal relevance | Individuals' perception on the relevance of SDM or of the specific implementation strategies for themselves or their professional group. | inductive | Major IF | Most participants perceived personal relevance of SDM and the study. | "Totally important. Precisely because it is so complicated and the patients cannot understand it at all. So who understands something like that, right? Somehow, even after six years of study, I find [it] super difficult and I think that's why it's so important to decide together. So I think it's very important." (interview I046, dept. 1, physician) | | 50 | 35 | 17 | 34,0 | 35 | 70,0 | rather barrier |
| | | | | | Some participants perceived the topic as less relevant to themselves.<br>- Junior physicians reported to not make decisions themselves, but be dependent on senior physicians.<br>- Some nurses see physicians as responsible for SDM implementation because physicians were more involved in decision-making.<br>- Nurses and physicians on inpatient wards: Decisions were more likely to be made in outpatient clinics, not on the wards. [cp. O3.2, O6.3] | "As a nurse, I have not yet dealt with this in such an extreme way, because it primarily concerns the physicians, because the physicians have to show the patients all these paths. And - and nurses can't really do that much." (interview I069, dept. 2, nurse) | | | | | | | | |
| | | | | | **SDM group training:** Some nurses did not perceive the training as relevant for themselves, but as more relevant for physicians. They voiced a lack of practice-orientation. | "She [a nurse] does not understand what the task of nurses is in the study. It was unclear to her why nurses had to be trained so intensively. From her point of view, intensive training makes sense for nurses in the wards, since they are very close to the patients. The nurses in the outpatient department are present during the examinations and the patient | | | | | | | | |

| | | | | | | | | | | | | | | |
|---|---|---|---|---|---|---|---|---|---|---|---|---|---|---|
| | | | | | | discussions, but they only provide assistance and are not involved in the decision-making process." (field note, dept. 3) | | | | | | | | |
| | | | | | **Ask3Q:** Postcards should be distributed by nurses rather than physicians. | "But I also think that this is actually more the task of the admitting nursing staff when the [patients] come. [...] To say, 'Here, we have this [the Ask3Q postcards]'." (interview I027, dept. 1, physician) | | | | | | | | |
| **I2** | **Self-efficacy** | CFIR: "Individual belief in their own capabilities to execute courses of action to achieve implementation goals." | CFIR | Mid-level IF | Some participants do not perceive themselves as leading medical decision-making processes.<br>- Senior physicians tend to lead decision-making processes, junior physicians and nurses tend to follow their lead.<br>- Thus, SDM implementation and participation in PREPARED study was reported to be dependent on head personnel's attitudes towards SDM. | "There would be difficulties with hierarchies; as a resident, it would be difficult to advise the patient in a conversation to weigh his decisions when the senior physician had already planned the treatment." (field note, dept. 2) | I2 and O2.1 are closely connected. All relevant statements were double coded with both codes. | 39 | 33 | 6 | 15,4 | 36 | 92,3 | rather barrier |
| **I3** | **Individual stage of change** | CFIR: "Characterization of the phase an individual is in, as he or she progresses toward skilled, enthusiastic, and sustained use of the intervention." | CFIR | No IF | One coding in total where a physician was already well versed in SDM. | "The team training is only attended by one physician [...]. She is very interested and seems to be very familiar with the topic of SDM. She says that she is already implementing many of it." (field note, dept. 1) | | 1 | 1 | 1 | 100,0 | 0 | 0,0 | rather facilitator |
| I3.1 | Individual motivation to change | Individuals' willingness to change their behavior towards more SDM. Including openness to feedback and learning. | inductive | Major IF | Motivation to change was perceived to vary greatly between individuals.<br>- Reported perception of colleagues: Some will change their behavior, others do not want to change.<br>- Some participants, however, see potential for change in themselves. | "So certainly that's when you've been around and working for a long time, I can imagine that it can already be food for thought." (interview I031, dept. 1, physician) | | 62 | 27 | 51 | 82,3 | 14 | 22,6 | rather facilitator |
| | | | | | Motivation to change was reported to have an impact on study participation.- People who were already interested in SDM would be more likely to take part in group training.- People who think they already do SDM were perceived less likely to be motivated to participate in study and change. | "But it was also noticeable that those who took part in the training were generally interested in changing something a bit. Yes, and those who I would judge myself in principle to need it more, unfortunately did not take part in the training." (interview I007, dept. 1, nurse in leadership position) | | | | | | | | |
| | | | | | **Coaching**: Many physicians saw at least the possibility to change a little towards more SDM. Feedback was for the most part well received. | "The physician particularly liked the tip to visualize the different treatment options on a sheet of paper - she never does this before, but finds it helpful and easy to implement and would like to try it out in subsequent discussions." (field note, dept. 1) | | | | | | | | |
| | | | | | **MDTMs**: Changes in MDTMs were reported to be difficult because of the lack of motivation to change of some individual participants and team dynamics. | "Overall, [a senior physician] appears genuinely interested and tells me again that she thinks [tumor boards] are difficult to change because a lot of it is up to the characters who sit on the [tumor boards]." (field note, dept. 1) | | | | | | | | |
| **I4** | **Individual identification with organization** | CFIR: "A broad construct related to how individuals perceive the organization, and their relationship and degree of commitment with that organization." | CFIR | No IF | No codings | No quotes | | 0 | 0 | | | | | |

| | | | | | | | | | | | | | | |
|---|---|---|---|---|---|---|---|---|---|---|---|---|---|---|
| **I5** | **Other personal attributes** | CFIR: "A broad construct to include other personal traits such as tolerance of ambiguity, intellectual ability, motivation, values, competence, capacity, and learning style." | CFIR | Minor IF | Structural reasons for non-participation in PREPARED study and its implementation strategies<br>- Career starters reported that other aspects of their work had priority.<br>- Priorities for people primarily working in research at the time reported other priorities.<br>- Part-time work was reported to make participation in trainings impossible. | "At the moment, I'm still so busy with training that we haven't really been able to deal with it yet." (interview I095, dept. 1, nurse) | | 34 | 25 | 11 | 32,4 | 23 | 67,6 | balanced |
| | | | | | Individual characteristics were reported to influence SDM uptake:<br>- Psychological stability: Sealing with patients' questions and concerns was reported to require psychological strength in nurses. Reportedly, not everyone can deal with it.<br>- Younger colleagues were reported to be more insecure.<br>- High personal burden by one's profession was reported to impede SDM.<br>- Specialization of physicians: Some specialized physicians were perceived as more open to SDM than others.<br>- Some nurses reported little interest in additional continuing medical education.<br>- Limited cognitive capacity: Physicians cannot remember all of the potentially available therapy options in detail. | "It is very difficult for the individual physician to know every possible therapy option and the resulting long-term risks and to discuss them optimally with the patient. This requires an immense amount of detailed knowledge, which cannot be assumed by all physicians off the cuff." (field note, no specific dept.) | | | | | | | | |
| **O** | **Inner setting** | | | | | | | | | | | | | |
| **O1** | **Organizational leadership** | Scoping review (SR): "Degree to which the management of a department or hospital supports the intervention (i.e. SDM)." | SR | | No codings in this superordinate category | No quotes | | 0 | 0 | | | | | |
| O1.1 | Corporate mission and vision statement | SR: "Degree to which the description of the organization's core purpose and vision for the future supports SDM." | SR | Minor IF | Several statements that SDM and the PREPARED study are supported at hospital.<br>- The hospital was seen to support research in general.<br>- The cancer center was reported to have SDM and patient-centeredness as a focus.<br>- Involvement of nurses in decision-making situations was reported to be desired. | "In doing so, it became clear to me that [the cancer center] has named patient-centeredness and shared decision-making as one of several focal points, and the PREPARED and ASPIRED projects [i.e., two projects from the workgroup] are named and outlined there." (field note, no specific dept.) | | 5 | 5 | 4 | 80,0 | 1 | 20,0 | rather facilitator |
| | | | | | One statement that SDM was not in the focus of the hospital. | "I think the big problem is simply that it hasn't been as present thematically in [the hospital] so far." (interview I053, dept. 1, physician) | | | | | | | | |
| O1.2 | Encourageme nt / leadership engagement* | SR: "Degree to which leaders in organization proactively support SDM." / CFIR: "Commitment, involvement, and accountability of leaders and managers with the implementation." | CFIR, SR | Major IF | Support from HCPs in leadership positions was reported to vary between departments and different divisions of the departments. | "At the end of the training, [a ward manager] said that SDM was the topic of the future and therefore extremely important. Patients would want to participate more and more in decision-making, and practitioners must deal with this and be prepared for it." (field note, dept. 2) | | 78 | 43 | 46 | 59,0 | 30 | 38,5 | balanced |
| | | | | | It was said to be helpful when HCPs in leadership positions- talk positively about the study,- motivate to participate in emails,- drive the implementation of the implementation strategies,- announce training sessions as mandatory (positive impact on participation rates, impact on motivation of participants during the training is unclear and should be further evaluated), and-establish distribution of Ask3Q postcards in routine care delivery. | "[A senior physician] also makes it clear that it would be very helpful if [the chief physician] would send an e-mail stating that all physicians are obligated to participate in the team training. Here, it would be necessary to involve the management even more and to exert pressure from above." (field note, dept. 1) | | | | | | | | |

| | | | | | | | | | | | | | | |
|---|---|---|---|---|---|---|---|---|---|---|---|---|---|---|
| | | | | | Further requests to senior physicians:<br>- Training sessions should be during working hours, but participants should be exempted from service duties for the time of the training.<br>- Senior physicians should act as role models.<br>- Senior physicians should appreciate junior physicians for using SDM. | "So I think you just have to [exemplify] from the top down. And I think that the culture doesn't exist. I would also say that none of our senior physicians [participated in the training]." (interview I053, dept. 1, physician) | | | | | | | | |
| | | | | | **MDTMs**: Decision-making in MDTMs was perceived to be influenced by the presence of the departments' head physicians. | "At the end [of the tumor board], the head of surgery approaches me and says that the function "clinical director" is missing at the selection of the function [on the newly developed tumor board checklist]. The decision-making in the tumor board would be very different if the clinic director was present." (field note, dept. 1) | | | | | | | | |
| O1.3 | Performance measurement and feedback | SR: "Use of results of performance measurement or quality indicator metrics to indicate room for improvement." | SR | No IF | **MDTMs**: One coding in total where skepticism was voiced whether the use of the MDTM checklist has a benefit. | "Both [attending chief and senior physicians, respectively] show rather skepticism whether the use of the checklist could have an effect or benefit, but are open to pilot testing in the tumor boards of [their departments]." (field note, dept. 2) | | 1 | 1 | 0 | 0,0 | 1 | 100,0 | rather barrier |
| **O2** | **Organizational culture** | SR: "Degree to which an organization's culture supports SDM." / CFIR: "Norms, values, and basic assumptions of a given organization." | CFIR, SR | | No codings in this superordinate category | No quotes | | 0 | | | | | | |
| O2.1 | Autonomy of staff | SR: "Degree of flexibility that healthcare providers (HCPs) have to achieve organizational goals." | SR | Mid-level IF | SDM implementation and participation in PREPARED study was reported to be dependent on head personnel's attitudes towards SDM due to the hierarchical structure of health care delivery. | see I2 for quotes | I2 and O2.1 are closely connected. All relevant statements were double coded with both codes. | 39 | 32 | 5 | 12,8 | 35 | 89,7 | rather barrier |
| O2.2 | Shared views and goals | SR: "Degree to which team members share the same views and goals." | SR | No IF | - Patient-centeredness was sometimes perceived of secondary importance in this maximum care hospital.<br>- Senior physicians in department 3 appealed for common goals regarding SDM implementation.<br>- Physicians and nurses were reported to sometimes differ in their views regarding the "right" treatment. | "But the approach here is a completely different one, not to say that we do - well, that sounds stupid now - we do everything that the patients say, but the approach is rather to do everything from the university that others no longer do." (interview I077, dept. 2, nurse) | | 6 | 6 | 3 | 50,0 | 3 | 50,0 | balanced |
| O2.3 | Learning climate** | CFIR: "A climate in which: a) leaders express their own fallibility and need for team members' assistance and input; b) team members feel that they are essential, valued, and knowledgeable partners in the change process; c) individuals feel psychologically safe to try new methods; and d) there is sufficient time and space for reflective thinking and evaluation." | CFIR | Minor IF | A good learning climate in the clinics was emphasized by some participants. | "He [the coached physician] considers feedback on interviewing to be very important, especially for surgeons, because you never stop learning. He thought it would be good to be mirrored even after many years of professional experience. The colleagues at MKG would also always take our feedback in the coaching sessions to heart." (field note, dept. 2) | | 6 | 5 | 5 | 83,3 | 2 | 33,3 | rather facilitator |
| **O3** | **Organizational teamwork** | SR: "Degree to which a departments'/hospitals' teamwork supports or hinders SDM." | SR | | No codings in this superordinate category | No quotes | | 0 | | | | | | |

| | | | | | | | | | | | | | | |
|---|---|---|---|---|---|---|---|---|---|---|---|---|---|---|
| O3.1 | Attributions to other professional groups | Assumptions about another professional group (nurses about physicians or physicians about nurses) regarding their behavior regarding SDM, and assumed attitudes towards SDM. | inductive | No IF | Mainly statements by nurses about physicians:<br>- Nurses tended to see the responsibility for SDM with physicians, not with themselves.<br>- Some nurses questioned some physicians' uptake of SDM and patient-centeredness.<br>- Nurses perceived that physicians should participate in SDM training and coaching, but some nurses questioned physicians' willingness to do so. | "With physicians - they need it, I think, even more urgently than we do." (interview I082, dept. 2, nurse) | | 25 | 18 | 2 | 8,0 | 24 | 96,0 | rather barrier |
| O3.2 | Networks and communication & Coordination of care | CFIR (networks and communication): "The nature and quality of webs of social networks and the nature and quality of formal and informal communications within an organization." // SR (communication): "How information is shared within and between teams." // SR (coordination of care): "Deliberate organization of care by HCPs from different specialties." | CFIR, SR | Major IF | Communication and information exchange were reported to differ depending on setting (inpatient ward vs. outpatient clinic). | "That is - it would be the case that we would have to be present at the physicians' meetings and that the three groups of patient, nurse and physician would have to be involved somehow. But that is almost not possible here in the process. It's probably more possible on the wards, but it's difficult here." (interview I042, dept. 1, nurse) | | 58 | 35 | 7 | 12,1 | 51 | 87,9 | rather barrier |
| | | | | | Communication difficulties- Communication between HCPs was generally perceived as poor.- Many personnel changes would lead to communication difficulties.- Physicians after longer absence (e.g. because of clinical rotation to another department) found it difficult to receive information about the study. | "But as I said it is, probably also the labor turnover, e.g. But, as I said, it's a bit of a shame, because the transfer, I think, also the understanding for each other [nurses and physicians],[...] is then also always a bit on the line, because then mostly nurses speak the same language." (interview I032, dept. 1, nurse) | | | | | | | | |
| | | | | | Communication flow:<br>- Patients would raise different issues with nurses than with physicians.<br>- Decisions were reported to be made in outpatient setting prior to admission, less on wards. [cp. I1.2, O6.3]<br>- Nurses considered it inappropriate to perform pre-treatment consultations and informed consent during ward rounds.<br>- Nurses reported that patients were sometimes not aware of all consequences of surgery due to inadequate information. | "Often questions are more likely to be directed to nurses, in part because patients are not well educated and nurses have much closer contact with patients than physicians." (field note, dept. 2) | | | | | | | | |
| *O3.2.1* | *Interdisciplinary cooperation* | The nature and quality of teamwork between different professional groups (e.g. ward rounds or meetings with physicians and nurses). | inductive | Major IF | A lack of interdisciplinary exchange was reported:<br>- A desire for joint explanations by physicians and nurses was voiced, but perceived as hardly feasible due to organizational structures and lack of time.<br>- Only few joint rounds, hardly any existing organizational structures for interdisciplinary exchange.<br>- Nurses did not feel involved in decision-making situations, but would like to be more involved.<br>- Nurses reported to often get information only during or after ward rounds and then have to react to patients' reactions. | "Interdisciplinary multiplier training is not a problem, interdisciplinary team training is difficult because there are hardly any existing structures for interdisciplinary exchange (hardly any points of contact between nurses and surgeons)." (field note, dept. 2) | | 45 | 25 | 10 | 22,2 | 35 | 77,8 | rather barrier |
| | | | | | **SDM group training**: The need for interdisciplinary trainings became clear. | "So, as I said, I just think that part of it would be to really somehow get the training to take place together [with nurses and physicians], because then, I think, the dialogue is simply quite different." (interview I032, dept. 1, nurse) | | | | | | | | |
| **O4** | **Organizational resources*** | SR: "Availability of resources." // CFIR (available resources): "The level of resources dedicated for implementation and on-going operations including money, training, education, physical space, and time." | CFIR, SR | | No codings in this superordinate category | No quotes | | 0 | | | | | | |

| | | | | | | | | | | | | | | |
|---|---|---|---|---|---|---|---|---|---|---|---|---|---|---|
| O4.1 | Workload | The workload that HCPs face in their daily business. If workload is high, capacities for additional tasks or efforts might be limited. | inductive | Major IF | High workload was named as a barrier to SDM in all departments and divisions.<br>- High workload was reported as a barrier to SDM implementation in general.<br>- High workload was described as a barrier to participation in the study (e.g. regarding participation in training or coaching and distribution of information materials). | "One of the [training participants] states that the length and comprehensiveness of the decision-making talks depends very much on the appointments he has afterwards. He notes that he is often unable to conduct such discussions as he would like due to other appointments." (field note, dept. 1) | O4.1, O4.2, O4.5 are strongly connected. | 24 | 18 | 1 | 4,2 | 24 | 100,0 | rather barrier |
| *O4.1.1* | *Parallel trials* | The realization of more than one (or multiple) studies/trials in one department/ward/clinic. This might limit capacities to keep track of and engage in these studies. | inductive | Minor IF | High workload was reported to be caused in part by many studies running in parallel.<br>- Hence, the relevance for participation in a study must be clear. Also<br>- Studies running in parallel have to be well coordinated by the study teams. | "[A nurse in leadership position] describes that there is a lot of pressure on the wards and that many studies are currently being presented to the staff. She therefore needs to be well informed about the studies in order to be able to pass this information on to the staff and motivate them to cooperate." (field note, dept. 1) | | 7 | 5 | 0 | 0,0 | 7 | 100,0 | rather barrier |
| O4.2 | Time | SR: "Amount of time HCPs have per patient / patient visit / for SDM implementation." | SR | Major IF | Lack of time was named as a barrier to SDM in all departments and divisions. | "But I don't think you can improve anything structurally now, I think the crucial thing for shared decision-making is that you have time as a physician and also as a senior physician to discuss this with the patient." (interview I031, dept. 1, physician) | O4.1, O4.2, O4.5 are strongly connected. | 64 | 44 | 10 | 15,6 | 54 | 84,4 | rather barrier |
| | | | | | Lack of time was reported to impact patient consultations:<br>- Consultations could not be conducted in peace and quiet.<br>- Decisions were reported to have to be made quickly. However, patients should have the opportunity to reflect on decisions.<br>- Lack of time was reported to lead to more paternalistic physician behavior (i.e. taking the lead). | "Addressing the issue of "asking for questions", the physician said that it was partly intentional/wanted that she did not encourage the patients to have longer conversations, as there was no time for that. The nursing staff wanted to finish their rounds at 10 a.m. on time, and she herself had a lot to do. Extensive discussions are not planned." (field note, dept. 1) | | | | | | | | |
| | | | | | Lack of time was reported to impact implementation of the study's implementation strategies.**SDM group training**: [cp. O6.3]- Difficulty to schedule training sessions into the daily routine of the ward. - Sometimes training sessions' duration had to be shortened due to organizational structures at the departments.- Six-hours train-the-trainer sessions were reported to be difficult to incorporate.- For most participants duration of training sessions was appropriate, few participants perceived it as too long or too short. | "In general, [a senior physician] clarifies again that they could not seek changes that needed more time. They would not have the capacity for this, but he thinks that the proposed changes [for tumor board organization] could be implemented without more time." (field note, dept. 3) | | | | | | | | |
| O4.3 | Financial resources | SR: "Amount of money available for certain activities (here SDM implementation) within organization." | SR | Minor IF | More lucrative payment for certain treatments was reported to lead to non-neutral presentation of options. | "But at the end of the day, of course, you are a commercial enterprise and - well, I very rarely experience that a physician says, okay, well, I would think about it in your place and quite often afterwards, I have already spoken with some [...] that they said, "well, if I had to make the decision alone, if I had something to say here, I would not have operated on him. But I had to because this gets money. The clinic wants to earn money somewhere and, let's say, fill the beds, and of course that doesn't work if people decide against [treatment] and leave." (interview I077, dept. 2, nurse) | | 4 | 3 | 0 | 0,0 | 4 | 100,0 | rather barrier |

| | | | | | | | | | | | | | | |
|---|---|---|---|---|---|---|---|---|---|---|---|---|---|---|
| O4.4 | Space | SR: "Amount of physical space available for certain activities (here SDM implementation) within organization." | SR | Minor IF | Lack of space was reported for some areas.<br>- Seating arrangement in consultation rooms of the outpatient clinic of department 2 was perceived as hindering good physician-patient communication.<br>- Lack of space was reported to prevent display of patient information material and Ask3Q postcards and posters. | "The change in seating arrangements in the treatment rooms also seems to be particularly relevant for him. The role play in the training also made him aware of how important it is to sit at the same table as the patients for decision-making discussions. This is usually not the case at present, he said. Patients usually sit on a treatment chair, which has a very negative connotation and is scary per se." (field note, dept. 2) | | 21 | 9 | 0 | 0,0 | 21 | 100,0 | rather barrier |
| | | | | | Hygiene and fire safety regulations influenced the display of Ask3Q and information materials. | "However, the [senior physician and ward physician] state that information should be placed in the physician's rooms at most very sparingly and that hygiene and fire safety regulations must be observed." (field note, dept. 1) | | | | | | | | |
| O4.5 | Workforce | SR: "Availability and assignment of employees for certain activities (here SDM implementation) within organization (i.e. personnel resources)" | SR | Major IF | Staff shortage was reported as barrier to SDM implementation in general and to the implementation of the study's implementation strategies in particular. | "[...] we had or still have such a severe staff shortage that there is hardly anyone who could participate. And that's why we said we'd postpone it until the new year." (interview I089, dept. 1, nurse) | O4.1, O4.2, O4.5 are strongly connected. | 21 | 15 | 1 | 4,8 | 20 | 95,2 | rather barrier |
| **O5** | **Organizational priorities / relative priority**** | SR (organizational priority): "Degree to which other aspects of care delivery conflict or align with SDM." // CFIR (relative priority): "Individuals' shared perception of the importance of the implementation within the organization." | CFIR, SR | Major IF | The hospital was perceived by some participants to rather prioritize economic or research interests over patients' needs.<br>- Participants reported that in some situation, certain treatment options would be prioritized by physicians in leadership positions (given equal evidence). | "But they [physicians] didn't let us [nurses] talk to them, [...] but somehow it came across as - hey, this is a big operation and you can do it quite well here, it's like a study object." (interview I082, dept. 2, nurse) | O5, S1.1, S3 are strongly connected. | 16 | 14 | 1 | 6,3 | 15 | 93,8 | rather barrier |
| | | | | | Patients were assumed to have other priorities and not pay attention to interventions like Ask3Q. | "The [patients] don't even have an eye for it [...] - they have completely different problems, worries, needs and fears." (interview I008, dept. 1, nurse) | | | | | | | | |
| **O6** | **Organizational workflows** | SR: "Degree to which a departments'/hospitals' workflows support or hinder SDM." | SR | | No codings in this superordinate category | No quotes | | 0 | | | | | | |
| O6.1 | Patient information dissemination strategies | SR: "A broad construct related to the availability of methods to disseminate information to patients and compatibility of workflows with decision aid distribution processes." // This includes for example statements about proactive dissemination of information materials to patients by HCPs, about the "right timing" of information provision, and about the burden of too much information provision. | SR | Major IF | Statements on distribution of information materials in general:<br>- Distribution of information materials was reported to be the responsibility of nurses. It was perceived to be already done on a routine basis (albeit variation between different divisions of departments). [cp. I1.1]<br>- Nurses reported to encourage patients to ask questions during ward rounds, but patients would often not do so. [cp. I1.1]<br>- Inclusion of patient relatives was reported to be also important. Patient relatives should also receive information. | "Then we always say, yes, ask the physician or talk it over again with the physician beforehand. Then they usually don't do it because they are totally nervous and forget half of it. Where I always say, make yourself a note that you work off with the physician. That helps the best." (interview I055, dept. 1, nurse) | | 80 | 48 | 44 | 55,0 | 38 | 47,5 | balanced |
| | | | | | **Ask3Q**:- Few participants expressed that Ask3Q would not be sufficiently noticed and taken up by patients.- Nursing professionals found it useful to incorporate Ask3Q into routine processes. In some clinical divisions within the study, postcards were routinely distributed.- Ask3Q postcards were more often distributed by nurses than by physicians. However, nurses saw it as the physicians' task to distribute the cards.- It was considered important to explicitly make patients aware of the postcards and posters. If they would only be displayed, they would not be noticed enough.- Postcards should be distributed at the time of admission or in the outpatient clinic.- Cards should be available in the patients' rooms. Patients would not perceive the | "The first contact is in the outpatient clinic. So it makes most sense, it has to be said, in our outpatient clinic here, that the patients are given the [Ask 3 Questions] perhaps right from the start. So rather - that has to be improved in the outpatient department." (interview I068, dept. 2, physician) | | | | | | | | |

| | | | | | | | | | | | | | | |
|---|---|---|---|---|---|---|---|---|---|---|---|---|---|---|
| | | | | | information if it could only be found in the corridors (e.g. at the counter). | | | | | | | | | |
| O6.2 | Electronic health record (EHR) | SR: "Availability of an EHR to be used in SDM (e.g. documentation of process)." | SR | No IF | No codings | No quotes | | 0 | | | | | | |
| O6.3 | Compatibility** & Scheduling routines and timeframes | CFIR (compatibility): "The degree of tangible fit between meaning and values attached to the intervention by involved individuals, how those align with individuals' own norms, values, and perceived risks and needs, and how the intervention fits with existing workflows and systems." // SR (scheduling routines and timeframes): "Degree to which scheduling (e.g. of appointments or for procedures) and time frame available until decision is needed impacts SDM." | CFIR, SR | Major IF | Organization of patient care and treatment processes was perceived not compatible with SDM.<br>- Timing of patient appointments would prevent the presentation of more than one option and longer consultations. [cp. O4.2]<br>- Decisions were reported to be predominantly made in outpatient clinics, not on wards (especially in department 2). Patients would sometimes only come to the ward after surgery. Thus, SDM needs to be implemented in outpatient clinics. [cp. I1.2, O3.2] | "So with the patients here now on the ward, who are somehow here first for diagnostics or come as an emergency and were not operated on directly, I think it [distributing Ask3Q] is an option, because of course you can still talk to them before they are informed about the surgery. Otherwise, the other patients who also receive same day surgery, for example, are usually informed downstairs in the outpatient clinic. Then, you would have to distribute the first piece of paper [Ask3Q] there." (interview I062, dept. 2, nurse) | This code was predominantly used to describe structural/organizational compatibility or incompatibility. // O6.3, P3.2, P3.3 are strongly connected. | 71 | 47 | 17 | 23,9 | 54 | 76,1 | rather barrier |
| | | | | | **SDM group trainings:** [cp. O4, P3.3]<br>- Routine clinical work was perceived to impede participation in trainings. High workload was reported to make participation impossible.<br>- Training sessions would need to be scheduled far ahead of time as rosters are written more than two months in advance. Only with early planning could training sessions be integrated into regular staff meetings or staff be scheduled out for participation.<br>- Some participants did not deem it possible to organize for nurses to attend SDM training during working hours. Nurses would have work overtime or participate off duty. | "Participation in the multiplier training will generally also be difficult at other times due to very limited time resources. The implementation of the team training is also difficult, as only a maximum time window of 45 minutes will be possible for this; in the normal working day, a longer training is not feasible - not even for the nursing staff alone." (field note, dept. 1) | | | | | | | | |
| | | | | | **Ask3Q**: Implementation of Ask3Q in clinical processes was considered possible by some participants. | Interviewer: "Would that [the Ask3Q intervention] be something where you say, I could imagine [to use it]. If we would get that available then [...] that you would then integrate that [in working routines]?" Interviewee: **"** Yes. Absolutely." (interview I008, dept. 1, nurse) | | | | | | | | |
| *O6.3.1* | *Reliability and predictability* | Cooperation partners' and participants' reliability regarding e.g. appointments or required feedback and its impact on the execution of the implementation program. This includes for example short notice cancellations of coaching sessions due to | inductive | Major IF | **SDM group trainings, MDTM reflection meetings, and study coordination meetings**:<br>- Cooperation partners and participants did sometimes not respond to the study team's emails or remained vague, so that appointments did not take place or had to be rescheduled.<br>- (Registered) participants for meetings and training sessions did not show up.<br>- Group training dates were canceled at short notice due to high workload. | "There were 8 people signed up for the meeting [reflection on MDTM organization], one person indicated he might come. Only 3 persons from [department 1] appeared (including the person who said he might come)." (field note, dept. 1) | | 71 | 19 | 5 | 7,0 | 66 | 93,0 | rather barrier |

| | | | | | | | | | | | | | | |
|---|---|---|---|---|---|---|---|---|---|---|---|---|---|---|
| | | non-predictable clinical tasks. | | | **Coaching:**<br>Content of patient-physician consultations could not be pre-planned and was therefore sometimes less suitable for SDM feedback. Then, only feedback on general communication skills could be given.<br>- Especially on inpatient wards, consultations were reported to be difficult to plan ahead.<br>- The content discussed during the majority of follow-up appointments led to feedback on general communication skills rather than SDM.<br><br>Coaching appointments had to be arranged spontaneously due to clinical processes or were spontaneously postponed by the physicians, because [cp. O4.1, O6.3]<br>- physicians had to take on other tasks than planned at short notice,<br>- physicians did not expect suitable consultations for SDM,<br>- physicians did not know when they would talk to the patients,<br>- physicians had too much other work to do, or<br>- patients cancelled at short notice or did not show up. | "It's - it's just - you always don't know where you're assigned until the next day and then to remember to let you know, that just makes it a little bit difficult then." (interview I080, dept. 2, physician) | | | | | | | | |
| *O6.3.2* | *Personnel changes* | Fluctuation in personnel on wards and in clinics due to e.g. planned rotations to other departments, new hires, and terminations. These include head personnel as well as junior positions. | inductive | Minor IF | - High staff fluctuation in some divisions lead to much unrest in the teams, lack of contact persons for the study team and for patients. New colleagues did not receive information about the study or received it late.- Changes in leadership positions lead to unrest and restructuring in the departments. | "So in the outpatient clinic it's just - we have a lot of changing staff. A lot of throughput. And the conditions are very difficult overall anyway with the rooms here. All in all, it's very difficult. And every change here is very complex." (interview I083, dept. 2, physician) | | 26 | 18 | 0 | 0,0 | 26 | 100,0 | rather barrier |
| **O7** | **Cosmopolitanism*** ** | CFIR: "The degree to which an organization is networked with other external organizations." | CFIR | No IF | No codings | No quotes | | 0 | | | | | | |
| **O8** | **Patient needs and resources*** ** | CFIR: "The extent to which patient needs, as well as barriers and facilitators to meet those needs, are accurately known and prioritized by the organization." | CFIR | Mid-level IF | Variation in patient activation was reported.<br>- Some participants said that more and more patients wanted to participate in decision-making and were increasingly informed.<br>- In some divisions it was reported that patients had inhibitions about asking questions, did not trust physicians, and were overwhelmed by too much information.<br>- In other divisions (especially those with long treatment cycles and hospital stays), patients were perceived to often have many questions and a lot of prior knowledge about their own disease and to be able and willing to participate in decision-making. | "I think it's quite important because there are more and more - there are also more and more patients who want to participate in decision-making, who are also well informed. And also want to be informed. And I think that has increased, so felt are the - it's more about deciding together." (interview I098, dept. 3, physician) | | 22 | 18 | 11 | 50,0 | 11 | 50,0 | balanced |
| O8.1 | Patients reach out to nurses rather than physicians | Nurses' or physicians' perception that patients are more likely to talk to nurses about e.g. their questions, doubts, and/or emotions than to physicians. | inductive | No IF | Nurses were reported to sometimes receive more information from patients than physicians.<br>- Patients would rather tell concerns about treatments and other problems to nurses than to physicians.<br>- Nurses would inform physicians if patients still have questions. | "[A ward manager] was very interested during the training. He explained that the nursing staff on his ward make many decisions together with the patients and, above all, also support decision-making processes, for example by answering additional questions from the patient after a discussion with the physician or by "translating" what has been said once again in an understandable way." (field note, dept. 1) | | 12 | 12 | 9 | 75,0 | 4 | 33,3 | rather facilitator |

| | | | | | | | | | | | | | | |
|---|---|---|---|---|---|---|---|---|---|---|---|---|---|---|
| O8.2 | SDM applicability in certain situations/with certain patients | Applicability of SDM in certain situations or for patients with certain characteristics. | inductive | Major IF | SDM was perceived **not suitable for certain disease situations**.<br>- There would be only one available treatment option for certain cancer entities and disease situations (e.g. leukemia).<br>- SDM was perceived not suitable for clinical emergencies.<br>- SDM was perceived especially suitable for the palliative setting, less so for curative approaches. | "Here on the ward it is simply the case that for the diseases we have, namely only [tumor entity XY], there is basically only one therapy option and that the alternative is always to die and, well, simplified now, but nevertheless it is roughly like that and if you then have two therapy options, then there is of course little shared decision-making and since we usually have younger and fitter patients here, where it is not an issue at all, that they perhaps decide in favor of a palliative concept." (interview I031, dept. 1, physician) | | 89 | 49 | 12 | 13,5 | 80 | 89,9 | rather barrier |
| | | | | | SDM was perceived **not suitable for certain patients**.<br>- Some patients were reported to be overwhelmed and preoccupied with their emotions, especially shortly after diagnosis. Then they would have no capacity to participate in decision making or to receive informational materials or Ask3Q.<br>- Some patients were reported to rather be guided and leave the decision to physicians.<br>- SDM was perceived not suitable for physically severely impaired patients.<br>- Some patients perceived to be cognitively not capable for SDM.<br>- Information about too many options would confuse patients.<br>- Some patients were reported to have many questions, others do not.<br>- Sometimes patients would already have fixed opinions about treatment at the beginning of the consultation (especially in cancer care). | "And you notice that the older patients don't ask as many questions, but they want you to make the decision for them and recommend something clearly, and most of them comply." (interview I034, dept. 1, physician) | | | | | | | | |
| | | | | | SDM was perceived not to fit into clinical routines. [cp. O6.3] | "Patients often come from outpatient clinics, which means that the procedure has already been discussed with them. They often come for endoscopy, for example. [...] they usually have the procedure in the morning. As a ward physician, you don't start talking to them again about alternatives." (interview I053, dept. 1, physician) | | | | | | | | |
| O8.3 | Feedback from patients | Patients talk to their HCPs about the study, the implementation strategies, and/or SDM in general. This includes for example that patients give feedback that a certain intervention was helpful to them. | inductive | Minor IF | **Ask3Q**:- Nurses observed that patients would notice the Ask3Q posters and take the postcards with them.- Nurses reported to not be approached about Ask3Q by patients on a regular basis.- When nurses did talk to patients about Ask3Q, patients would perceive Ask3Q as helpful and feel taken more seriously as a result. | "They think it [the Ask3Q intervention] is all great! They have already given a lot of feedback that they think it's good." (interview I091, dept. 3, nurse) | | 29 | 26 | 22 | 75,9 | 8 | 27,6 | rather facilitator |
| **O9** | **Structural characteristics** | CFIR: "The social architecture, age, maturity, and size of an organization." | CFIR | No IF | This code includes various structural aspects.<br>- Nurses reported not to be involved in decision-making since there were no interdisciplinary or nursing ward rounds.<br>- Implementation of the study's implementation strategies was reported to be easier on wards than in outpatient clinics due to structural characteristics.<br>- In wards for privately insured patients, physicians were reported to have more time for consultations than in other wards.<br>- The need to clarify responsibilities for distribution and sorting of information materials was voiced. | "This is easier to realize in the inpatient clinic - it was. And that also went there, so to speak, much more clearly. [...] In the outpatient clinic [...] that is much, much more difficult than, as I said, in the inpatient clinic, the distances are apparently shorter." (interview I083, dept. 2, physician) | For the most part, contents seem to be already covered in other codes. | 18 | 17 | 6 | 33,3 | 12 | 66,7 | balanced |

| | | | | | | | | | | | | | | |
|---|---|---|---|---|---|---|---|---|---|---|---|---|---|---|
| | | | | | - For hygienic reasons (e.g. leukemia ward) no posters / postcards / information material can be distributed. | | | | | | | | | |
| O9.1 | Structural changes | Changes in department structures over the course of the study. This includes for example wards being relocated to another building/floor, renovations, or complete teams being newly put together. | inductive | Minor IF | Restructuring, merging, conversions, and renovations of wards (different departments were combined on one ward, wards moved within the building, new wards were formed) were reported as barriers to the implementation program. | "But the problem is that our ward is currently in a state of upheaval. Our ward manager has left because of pregnancy, and we only have an acting ward manager, many employees have just left and now there are vacancies and we are working with minimum staffing - that's just it, so we don't have the opportunity to deal with it more actively right now." (interview I026, dept. 1, nurses) | | 9 | 6 | 0 | 0,0 | 9 | 100,0 | rather barrier |
| **O10** | **Tension for change**** | CFIR: "The degree to which stakeholders perceive the current situation as intolerable or needing change." | CFIR | Minor IF | Motivation to initiate change was reported in many areas. However, especially in MDTMs, people tend to perceive change as unrealistic. | "Nurses in particular gave the impression that they saw room for improvement and was quite open to new aspects of the topic [SDM]." (field note, dept. 1) | Strong tension for change was interpreted as a facilitator for SDM implementation. | 17 | 13 | 10 | 58,8 | 9 | 52,9 | balanced |
| **O11** | **Organizational incentives and rewards**** | CFIR: "Extrinsic incentives such as goal-sharing awards, performance reviews, promotions, and raises in salary, and less tangible incentives such as increased stature or respect." | CFIR | No IF | One coding in total where a physician asked if she would be co-author of the study's publication because of her participation in the study as a multiplier. | "Towards the end of the training [for multipliers] (during the presentation of the multiplier concept), a physician, who was 1.5 hours late, asked what she got out of participating in the training (participation in publication?). We answer that this is not planned." (field note, dept. 1) | | 1 | 1 | 0 | 0,0 | 1 | 100,0 | rather barrier |
| **S** | **Outer setting** | | | | | | | | | | | | | |
| **S1** | **Incentives****** | SR: "Incentives implemented at the level of the health care system that impact SDM implementation." | SR | | No codings in this superordinate category | No quotes | | 0 | | | | | | |
| S1.1 | Payment model | SR: "Impact of payment models on the use of SDM. This includes e.g. possibilities to bill for SDM." | SR | Major IF | The hospital was perceived as a commercial enterprise.<br>- Long physician-patient consultations were reported to not be remunerated accordingly.<br>- Certain treatments that do not bring money were reported to not be discussed with patients in decision-making consultations. | "Well, I mean - that always sounds so harsh, doesn't it? But at the end of the day, of course, you're a business enterprise at the end of the day, and - well, I very rarely hear a physician say, okay, I'd think about that if I were you." (interview I045, dept. 3, nurse) | O5, S1.1, S3 are strongly connected. | 7 | 6 | 1 | 14,3 | 6 | 85,7 | rather barrier |
| S1.2 | Accreditation/ certification criteria | SR: "Degree to which SDM is included as a criterion in accreditation/certification standards for healthcare institutions." | SR | Mid-level IF | Study was presented and well received in certification audits of department 1 and 2. This was initiated by the clinical cooperation partners. | "We were - three days ago there was an audit of the oncological - well, oncological audit, they found it very praiseworthy. They are many - from many different cities and they thought it was really good, they also presented this study." (interview I077, dept. 2, nurse) | | 11 | 9 | 8 | 72,7 | 3 | 27,3 | rather facilitator |
| | | | | | **Ask3Q**: Some HCPs assumed a connection between the distribution of the materials and a simultaneous accreditation audit. | Interviewer: "Ok. Ever seen this postcard or flyer?" Interviewee: "Yes. Yes." Interviewer: "Yes? In what context?" Interviewee: "Oh, they were hung up all over the hallway here for onco-certification." (interview I014, dept. 1, nurse) | | | | | | | | |
| | | | | | **MDTMs**: Integration of patient's perspective in MDTM discussion and MDTM recommendation letter was questioned regarding its usefulness and perceived to contradict the German Cancer Society's guidelines for MDTMs. | "The purpose of tumor boards is that the patient's perspective does not play a major role, but that evidence-based guidelines are in the foreground. This is what the German Cancer Society demands [...]." (field note, dept. 2) | | | | | | | | |
| **S2** | **Policies and guidelines****** | SR: "Degree to which (health) policies and guidelines support the use of SDM." | SR | No IF | No codings | No quotes | | 0 | | | | | | |

| | | | | | | | | | | | | | | |
|---|---|---|---|---|---|---|---|---|---|---|---|---|---|---|
| S2.1 | Legislation | SR: “Degree to which state or national legislation requires the use of SDM /decision support.” | SR | No IF | No codings | No quotes | | 0 | | | | | | |
| S2.2 | Practice guidelines | SR: “Degree to which relevant practice guidelines support the use of SDM.” | SR | No IF | No codings | No quotes | | 0 | | | | | | |
| S2.3 | Quality indicators | SR: “Degree to which quality indicators support the use of SDM.” | SR | No IF | No codings | No quotes | | 0 | | | | | | |
| **S3** | Culture of health care delivery | SR: “Degree to which the culture of health care delivery supports SDM.” | SR | Major IF | The focus of patient care at the hospital was perceived to be on provision of maximum care and curative treatments.<br>- Type of surgery or treatment was perceived as a "political decision".<br>- Information provision and obtaining informed consent was reported to vary between departments and medical specializations. | "[A senior physician] notes that different mentalities prevail within the disciplines. Surgeons are very convinced of their procedures and inform patients primarily about the advantages of surgery. Radiotherapists are more reticent, informing patients about disadvantages. Oncologists are more in the middle." (field note, dept. 1) | O5, S1.1, S3 are strongly connected. | 11 | 11 | 4 | 36,4 | 7 | 63,6 | balanced |
| | | | | | **Ask3Q**: Parallel to our study, an initiative "3 questions for your health" (very similar to Ask3Q) was launched by the city of Hamburg. On the instruction of the head physicians of department 1 and 2, the initiatives' materials were also distributed in the departments. | "In a conversation with [nurse in leadership position] I learn that in the MKG the information sheets of the city of Hamburg '3 questions for your health' are in circulation. These had been passed on to everyone 'by the boss'." (field note, dept. 2) | | | | | | | | |
| **S4** | HCP education and licensing | SR: “Degree to which HCP initial and continuing education and licensing include SDM training.” | SR | Minor IF | The inclusion of SDM in medical education was found to have increased over the years.<br>- Younger physicians reported to have learned about SDM in their studies. By some participants, this was mentioned as a barrier to participate in our study, because they saw no need for additional training.<br>- Other physicians reported that communication skills training played a minor role in their studies. | "Especially now for the many young colleagues, where one has this also again and again in the study, this topic, shared decision-making, that there is perhaps not quite so much interest, because also the question is, what is there at all new and what should be taught there [...]." (interview I031, dept. 1, physician) | | 13 | 10 | 8 | 61,5 | 7 | 53,8 | balanced |
| **S5** | Peer pressure | CFIR: “Mimetic or competitive pressure to implement SDM; typically because most or other key peer or competing organizations have already implemented or are in a bid for a competitive edge.” | CFIR | No IF | No codings | No quotes | | 0 | | | | | | |
| **V** | **Characteristics of the intervention** | | | | | | | | | | | | | |
| **V1** | **Intervention source** | CFIR: “Perception of key stakeholders about whether the intervention is externally or internally developed.” | CFIR | | No codings | No quotes | | 0 | | | | | | |
| V1.1 | Role of multipliers | Tasks and responsibilities the multipliers (i.e. clinical stakeholder who received a 5-hour SDM train-the-trainer SDM training in order to support implementation at their departments) took on. | inductive | Major IF | **SDM group training**: Multipliers expressed discomfort with their role to teach their colleagues in SDM without being experts themselves and reported feeling overwhelmed.<br>- Generally, participation of multipliers in team training sessions was perceived as helpful to highlight the practical relevance and to motivate participants.<br>- Multipliers part in team training sessions varied greatly. Only a few took on the role of lecturers. | "And then it was also said that we should actually do the [team] training and I found that a bit difficult, because you learned that on this day, but you are not directly a professional in this area." (interview I062, dept. 2, nurse) | Multipliers were predominantly found to feel not sufficiently prepared to train colleagues in SDM. // V1.1, V1.2, P2 are strongly connected. | 19 | 8 | 9 | 47,4 | 10 | 52,6 | balanced |

| | | | | | | | | | | | | | | |
|---|---|---|---|---|---|---|---|---|---|---|---|---|---|---|
| V1.2 | Support by study team | Extent of support given by the study team to implement the intervention. This includes for example team trainings administered by the study team (instead of the multiplicators). | inductive | Major IF | **SDM group training**: Training sessions were driven by the study team.<br>- For some training sessions, there were no multipliers available and sessions had to be carried out by the study team alone. [cp. V1.1]<br>- Support from the study team in the trainings was perceived as helpful and necessary. | "[It was helpful] that we did it a little bit together. And a [study team member] was always there and then added to it. That also made us feel better. Before you forget any things, that was quite good." (interview I062, dept. 2, nurse) | V1.1, V1.2, P2 are strongly connected. | 7 | 7 | 4 | 57,1 | 3 | 42,9 | balanced |
| **V2** | **Evidence strength and quality** | CFIR: “Stakeholders’ perceptions of the quality and validity of evidence supporting the belief that the intervention will have desired outcomes.” | CFIR | No IF | Only three codings in total regarding Ask3Q and information materials:<br>- Nurses questioned whether Ask3Q was helpful for patients.<br>- Participants questioned whether some of the information materials were evidence-based and thus suitable for patients. | "But that is now a - so from the patient's point of view always, that there is now a hurdle to ask us, I think that is rather not the case, [...] I do not know the concept [of SDM] now, but I would not know now, how that should help the patients. So honestly." (interview I031, dept. 1, nurse) | This topic was not the focus of our interviews and might hence be underrepresented in this analysis. | 3 | 3 | 1 | 33,3 | 2 | 66,7 | balanced |
| **V3** | **Relative advantage** | CFIR: “Stakeholders’ perception of the advantage of implementing the intervention versus an alternative solution.” | CFIR | Major IF | Perception of relative advantage varied.- Other issues were considered more relevant.- SDM in general, the study, and its implementation strategies were perceived to be beneficial to patients.- HCPs expressed the need to be aware of the potential (personal) advantages the study offered in order to be motivated to participate. | "From the beginning, there was a bit of a problem, that the physicians understand from the outset what they can extract in terms of positive effects for the efficiency of everyday life, and that was a bit lacking. [... This study was] so to speak primarily perceived only as additional work [...]." (interview I094, dept. 3, physician) | | 24 | 20 | 5 | 20,8 | 19 | 79,2 | rather barrier |
| **V4** | **Adaptability** | CFIR: “The degree to which an intervention can be adapted, tailored, refined, or reinvented to meet local needs.” | CFIR | Major IF | **SDM group training**:<br>- Duration of training sessions was adapted to the capacities of the participants (e.g. shortening team training sessions, splitting up the train-the-trainer session into two shorter session in department 3). [cp. 6.3]<br>- Physicians who had not participated in a training session received the training materials personally from the study team during the first coaching session or via mail. | "Splitting the training into 2 x 2.5 hours results in attrition between the two dates (physicians attend only one of the two dates)." (field note, dept. 3) | | 17 | 10 | 5 | 29,4 | 12 | 70,6 | rather barrier |
| | | | | | **Coaching:** Timing of coaching sessions could not always be implemented as planned in the study protocol (e.g. postponing due to absences of physician or study team). [cp. O6.3.1] | "Since the physician will be traveling from the end of January to the end of February, we will do the second coaching already next week." (field note, dept. 3) | | | | | | | | |
| | | | | | **Ask3Q and information materials**:<br>- One ward decided not to hang up Ask3Q posters and only distributed postcards due to overcrowding of walls.<br>- One ward at department 2 developed a welcome folder for patients on the basis of our study materials. | "In addition, I was told about a folder that they have made independently for the patient rooms, where the oncological patients can inform themselves. In front there was directly our poster in DINA4 and there is the postcard in it, further back then there is various information from the German Cancer Aid." (field note, dept. 2) | | | | | | | | |
| **V5** | **Trialability** | CFIR: “The ability to test the intervention on a small scale in the organization, and to be able to reverse course (undo implementation) if warranted.” | CFIR | No IF | No codings | No quotes | | 0 | | | | | | |
| **V6** | **Complexity** | CFIR: “Perceived difficulty of implementation, reflected by duration, scope, radicalness, disruptiveness, centrality, and intricacy and number | CFIR | Minor IF | Two codings in total:<br>- Therapeutic options nowadays were perceived as too complex to know all details by heart.<br>- The distribution of Ask3Q postcards was perceived to be difficult. Thus, it would have to be well prepared and organized. | "The question is whether patients can be informed about the situation after surgery in such a way that they can actually imagine what it will be like." (field note, dept. 2) | For the most part, contents seem to be already covered in other codes. | 2 | 2 | 0 | 0,0 | 2 | 100,0 | rather barrier |

| | | | | | | | | | | | | | | |
|---|---|---|---|---|---|---|---|---|---|---|---|---|---|---|
| | | of steps required to implement.” | | | | | | | | | | | | |
| V6.1 | Translation into practice | Degree to which the intervention or parts of it are transferable to routine day-to-day care. | inductive | Major IF | Transfer into practice was perceived as difficult.<br>- SDM concept was considered too theoretical.<br>- Practical exercises to repeat what has been learned in theory were assumed to have the potential to improve SDM implementation in practice.<br>- E.g. the implementation of Ask3Q was assumed to easily get lost in daily routine care if responsibility for the task was not clearly assigned to one person. | "I found the [SDM training] quite interesting, but I think for many of my colleagues and I it is very - in my opinion - a very theoretical concept, which I think is often not practically feasible. The idea is - a lot of it was correct and interesting - but I don't think it can be implemented one-to-one with our patients." (interview I024, dept. 1, physician) | | 14 | 11 | 4 | 28,6 | 10 | 71,4 | rather barrier |
| **V7** | **Design quality and packaging** | CFIR: “Perceived excellence in how the intervention is bundled, presented, and assembled.” | CFIR | Minor IF | Perception of Ask3Q design varied.<br>- Some found Ask3Q materials nicely designed and not cluttered.<br>- Others criticized the comprehensibility of the materials, lack of clarity about target audience, information sources, and in graphic design. | "And actually, I'm not a fool, but it was a bit opaque for me at first. Until it was explained to me. I mean the illustration." (interview I008, dept. 1, nurse) | | 35 | 31 | 20 | 57,1 | 16 | 45,7 | balanced |
| **V8** | **Cost** | CFIR: “Costs of the intervention and costs associated with implementing the intervention including investment, supply, and opportunity costs.” | CFIR | No IF | No codings | No quotes | | 0 | | | | | | |
| **V9** | **Access to knowledge and information*** | CFIR: “Ease of access to digestible information and knowledge about the intervention and how to incorporate it into work tasks.” // This includes knowledge and information about the study/intervention in general or specific implementation strategies. | CFIR | Major IF | Knowledge about the study:<br>- Some participants reported having received no information about the study or getting it too late.<br>- Some participants would have appreciated to be well informed before the study started.<br>- Some participants reported only seeing the Ask3Q materials on the wards, but missing additional information (e.g. brief presentation by the study team). | "The posters were simply there, the cards were there at some point, maybe it was announced somehow, that could be, but well. I somehow didn't notice anything about it and it was just these cards, they're just there." (interview I011, dept. 1, physician) | | 98 | 70 | 40 | 40,8 | 59 | 60,2 | balanced |
| | | | | | Information dissemination strategies in the study:- The study team provided information about the study and the individual components (e.g. through leaflets in common rooms, through short presentations in regular staff meetings, by mail or email). Several nurses and physicians reported that they had received information this information.- Information was passed on in regular staff meetings by the study's clinical cooperation partners and multipliers, but not all HCPs were always present at these meetings.- Persons returning to work after longer absences (e.g. parental leave, clinical rotations to other departments) reported a lack of information and had to proactively inform themselves. | Interviewer: "And what did you think of the fact that the colleagues [of the study team] came by and presented the whole thing?" Interviewee: "Yes, I thought that was good, because that is often the case, not that something is put up and then no one knows what to do with it. But then you just heard it, and I thought it was more personal, [...] and above all, you know who's walking down the hall here [regarding study team members], don't you?" (interview I021, dept. 1, nurse) | | | | | | | | |
| V9.1 | Reachability | Ways to reach participants/stakeholders and give them information. E.g. information on the study in general and its implementation strategies, scheduling of training and coaching via e-mail, | inductive | Major IF | Sometimes difficulties arose for the study team to reach clinical staff. | "There was generally very little response to e-mails. [A clinical cooperation partner] generally says on the subject of mails: 'No one responds to collective mails. More so when addressed personally.' As well as 'Emails are not really read.' Furthermore, she says in the course 'Nobody answers me anyway, nobody already has'." (field note, dept. 1) | | 85 | 67 | 51 | 60,0 | 35 | 41,2 | balanced |

| | | | | | | | | | | | | | | |
|---|---|---|---|---|---|---|---|---|---|---|---|---|---|---|
| | | superiors, team meetings, or newsletter. | | | Physicians<br>- Physicians received information reliably via email or through further regular staff meetings and continuing medical education meetings.<br>- But emails sometimes seemed to have gotten lost or physicians did not respond to inquiries. Being personally approached was perceived more binding. | "I think it's good [invitations to training courses and coaching sessions by e-mail] because you can then call it up again and again, so to speak, but it also gets lost a little bit because we always get a lot of [...] invitations to some things and of course you always read through it, but it gets lost a little bit. I think it would be good to have another one of those - I think there have already been once lunches [an internal training] and so on about it, right?" (interview I029, dept. 1, physician) | | | | | | | | |
| | | | | | Nurses<br>- Communication with the study team and reachability varied greatly between individual nurses in leadership positions.<br>- Nurses received information mainly from nurses in leadership positions in regular staff meetings, the minutes of these meetings, and via leaflets in the wards' common rooms. Occasionally, information was passed on by physicians.<br>- Nursing staff would like to receive information via individual mailboxes (often preferred option), via nurses in leadership positions, via the regular hospital-wide newsletters, via personal approach by the study team.<br>- Information provision via email was less preferred by nurses. | "I think that [information via e-mail] is quite good, although I know of many colleagues who never look in their e-mail box because they are not in any working groups or look in very irregularly, so I don't know if you can really reach all the staff well. I think a combination of e-mail and having something put in the post box again in person, so to speak, or depositing something on the ward, which then actually hangs somewhere, would be good." (interview I030, dept. 1, nurse) | | | | | | | | |
| V9.2 | Mandatory participation | Perception or communication by superiors that participation in e.g. training or coaching is mandatory. | inductive | Major IF | Participants perceived that the study's success depended on mandatory participation.<br>- Some nurses in leadership positions scheduled the training sessions as part of compulsory staff meetings or assigned all staff to a training session. [cp. O1.2]<br>- Some senior and head physicians announced participation in training sessions as mandatory. [cp. O1.2]<br>- However, participants reported that even in mandatory meetings not all physicians would participate.<br>- In department 2, physicians participating in the train-the-trainer session were selected by the head physician and obligated to participate. | "When implementing the Ask 3 Questions, we would have to consider how important it is that patients are given the cards directly. If this was presented to the physicians as an option, it would not be implemented. This would have to be made obligatory if it were to actually happen." (field note, dept. 3) | | 26 | 15 | 15 | 57,7 | 11 | 42,3 | balanced |
| V9.3 | Recurrence/ sustainability | Perception that several iterations of the implementation strategies might influence reach and possibly implementation outcome. | inductive | Minor IF | Few codings in total showing that regular refreshers about SDM and the study were perceived as helpful and that need for further training was perceived. | "So theoretically it wouldn't hurt to do something again. [...] Now, so to speak, after a while, maybe again - in the morning in the handover - to take this pocket cards and to say - by the way, remember again [to use them]. That's a good thing." (interview I092, dept. 3, physician) | | 4 | 4 | 2 | 50,0 | 2 | 50,0 | balanced |
| **P** | **Implementation process** | | | | | | | | | | | | | |
| **P1** | **Planning** | CFIR: "The degree to which a scheme or method of behavior and tasks for implementing an intervention are developed in advance, and the quality of those schemes or methods." | CFIR | No IF | No codings | No quotes | This category was not a focus of the data collection for this qualitative analysis. However, e.g. the pilot study for the development of the implementation program might have been major influencing factors. | 6 | 5 | 1 | 16,7 | 5 | 83,3 | rather barrier |

| | | | | | | | | | | | | | | |
|---|---|---|---|---|---|---|---|---|---|---|---|---|---|---|
| **P2** | **Engaging** | CFIR: “Attracting and involving appropriate individuals in the implementation and use of the intervention through a combined strategy of social marketing, education, role modeling, training, and other similar activities. // 1. Opinion Leaders: Individuals in an organization who have formal or informal influence on the attitudes and beliefs of their colleagues with respect to implementing the intervention. // 2. Formally Appointed Internal Implementation Leaders: Individuals from within the organization who have been formally appointed with responsibility for implementing an intervention as coordinator, project manager, team leader, or other similar role. // 3. Champions: Individuals who dedicate themselves to supporting, marketing, and ‘driving through’ an implementation, overcoming indifference or resistance that the intervention may provoke in an organization. // 4. External Change Agents: Individuals who are affiliated with an outside entity who formally influence or facilitate intervention decisions in a desirable direction.” | CFIR | Major IF | For the most part, clinical cooperation partners were described as very supportive.<br>However, some difficulties were described [cp. O6.3.1]:<br>- Some clinical cooperation partners were hard to reach.<br>- Participants did sometimes not show up for agreed appointments. Especially MDTM reflection meetings had to be postponed on several occasions.<br>- Sometimes previously discussed agreements were forgotten or not implemented. | "Both [clinical cooperation partner] and [clinical cooperation partner] as well as [senior physician from another deparment] were very supportive in the follow-up (e.g., quick feedback on the protocol, open to help us pilot the checklist)." (field note, dept. 3) | V1.1, V1.2, P2 are strongly connected. | 10 | 4 | 2 | 20,0 | 8 | 80,0 | rather barrier |
| **P3** | **Executing** | CFIR: “Carrying out or accomplishing the implementation according to plan.” | CFIR | Mid-level IF | Motivation and capability to engage in the study and its implementation strategies influenced execution.<br>- Some registered participants did not show up for training sessions.<br>- Role-playing in training sessions was perceived as important for successful learning and behavior change. However, motivation to role-play in train-the-trainer sessions was low.<br>- Integrating personal handovers of Ask3Q postcards and other information materials to patients into routine care routines was perceived as possible and executed by some participants and divisions, but not by others.<br>- Individuals being explicitly responsible for certain implementation strategies were reported to be helpful for successful implementation. | "But I also always have this - this piece of paper with the three questions [the Ask3Q postcard], [...] but to be honest I haven't used it that often. It's in front of me and somehow you forget it." (interview I092, dept. 3, physician) | For the most part, contents seem to be already covered in other codes. | 22 | 13 | 8 | 36,4 | 14 | 63,6 | balanced |

| | | | | | | | | | | | | | | |
|---|---|---|---|---|---|---|---|---|---|---|---|---|---|---|
| P3.1 | Too many approaches by study team | Participants‘ perception that the study team approached the potential participants (i.e. HCPs) too many times (e.g. sent too many e-mails). | inductive | Minor IF | Reminder emails sent by the study team were perceived as too many by some participants. Some participants reported to feel guilty about not responding or not having time to participate in training or coaching | Interviewee: "You feel a bit under pressure. [...] it's just a bit too much." Interviewer: "That means rather one e-mail less, one reminder e-mail less, right?" Interviewee: "Yes, I think you read it through more, because if you already - it's almost like a spam mail that you think, oh yes, PREPARED study." (interview I044, dept. 1, physician) | | 5 | 4 | 2 | 40,0 | 3 | 60,0 | balanced |
| P3.2 | Participation during working hours | Extent to which participation in the implementation strategies (i.e. training, coaching, meetings) took place during working hours. | inductive | Major IF | **SDM group training:** Participation during working hours was desired. [cp. O6.3]<br>- Training sessions frequently took place after HCPs' shifts in their free time. This was reported as a barrier due to exhaustion and low motivation.<br>- Duration of training sessions would have to be shortened, as current duration of training sessions was reported to be not possible during working hours. | "By chance, I also met [a physician cooperation partner], brief conversation about the trainings. Many of the physicians directly approached said that 5-hour training sessions were too much after work and that many were happy to finally call it a day. Most of them liked the cooperation as long as it was within their working hours." (field note, dept. 1) | O6.3, P3.2, P3.3 are strongly connected. | 15 | 14 | 3 | 20,0 | 11 | 73,3 | rather barrier |
| P3.3 | Integration into existing structures | Extent to which implementation strategies of the study (e.g. training, coaching, dissemination of information material) could be administered within existing structures (e.g. regular team meetings, existing information provision pathways). | inductive | Major IF | **SDM group training:** Embedding training sessions into existing structures was perceived as helpful. [cp. O6.3]- Training courses were frequently embedded in regular staff meetings of nurses or physicians.- Integration of training courses into regular staff meetings is sometimes difficult, as these are already filled with other topics.- Training sessions must not exceed the corresponding time frame of the regular meetings. Thus, some training sessions had to be shortened to 30-45 min. | "I think that's the problem with the appointments. I think the advantage is, of course, if you now have fixed appointments where everyone goes, onco-lunch [a regular internal training] or so, that then, I think, the participation would be somewhat higher, as if it is in the clinic everyday life. Then always something comes in between and then that is rather an appointment where you say, you skip it." (interview I056, dept. 1, physician) | O6.3, P3.2, P3.3 are strongly connected. | 17 | 11 | 7 | 41,2 | 9 | 52,9 | balanced |
| P3.4 | SARS-CoV-2 pandemic situation | Extent to which the SARS-CoV-2 pandemic situation might have influenced the uptake of SDM in general and the study’s implementation strategies in particular. | inductive | Minor IF | The SARS-CoV-2 pandemic started after the last implementation interval and during the last measurement wave of the study. This led to the following adaptations:<br>- Temporary recruitment stop for ten weeks during last measurement wave.<br>- Temporary stop of replenishment of Ask3Q and information materials. Subsequently, less Ask3Q and information materials were available for patients, assumably due to hygiene regulations.<br>- Seating arrangements in MDTMs was adjusted during the pandemic.<br>- MDTMs were conducted in the shortest possible time and with fewer participants during the pandemic Therefore, the patient perspective seemed to be less integrated. | "However, he [the chief physician] points out that the tumor boards are currently kept as short as possible and therefore there is even less opportunity to discuss the patient's perspective or wishes." (field note, dept. 1) | | 7 | 6 | 1 | 14,3 | 6 | 85,7 | rather barrier |
| P3.5 | Loss of materials (German: Schwund von Materialien) | Extent to which study materials (e.g. stand-up displays for information material, Ask3Q postcards and posters) went missing over the course of the study that could not be explained by the intended use of materials (unintended loss). | inductive | Minor IF | Display stands, Ask3Q postcards, and other study materials were no longer available or lost, possibly due to renovations or the SARS-CoV-2 pandemic. | "In the lounge of [an inpatient ward] we noticed that in the last two weeks the lounge was renovated, among other things furniture was replaced. In the course of this, we noticed that a display with postcards was missing." (field note, dept. 1) | | 10 | 5 | 0 | 0,0 | 10 | 100,0 | rather barrier |

| | | | | | | | | | | | | | |
|---|---|---|---|---|---|---|---|---|---|---|---|---|---|
| **P4** | **Reflecting and evaluating** | CFIR: “Quantitative and qualitative feedback about the progress and quality of implementation accompanied with regular personal and team debriefing about progress and experience.” | CFIR | No IF | No codings | No quotes | | 0 | | | | | |
| P4.1 | No changes noticeable /not helpful | Perception that the implementation program did not change routine care (i.e. did not have the anticipated effects) and/or perception that the implementation program was not helpful. | inductive | No IF | Some participants voiced disappointment about the study and its impact.<br>- Some participants stated that nothing had changed over the course of the study.<br>- Some nurses perceived the training as not helpful to them.<br>- In MDTMs, mostly no changes were perceived. | Interviewer: "Has anything changed in the conversations? [...]" Interviewee: "Well, I would say something. Even if I had hoped for more at the beginning, especially after the training. I have to say. I can't say that directly for the others. From the feeling not significantly much or sufficiently, I would say. But for me - well, I notice that especially in the tumor consultations. I would say that it has already had an influence on the patients. [...]" (interview I050, dept. 2, physician) | Perception that the trial did not lead to sufficient changes might have de-motivated for ongoing engagement in the study. | 9 | 8 | 2 | 22,2 | 8 | 88,9 | rather barrier |
| P4.2 | Implementation study perceived as helpful | Perception that the implementation program was helpful and/or participants being thankful for the implementation efforts of the study team. | inductive | No IF | Several participants perceived the study as helpful.<br>- Participants thanked the study team for conducting the study and good cooperation.<br>- Awareness of SDM was reported to have changed over the course of the study.<br>- Training and coaching sessions were described as helpful.<br>- Physicians were thankful for coaching feedbacks. | "She [a coached physician] also emphasized again that she and also colleagues had found the coaching very helpful." (field note, dept. 3) | Perception that the trial was helpful might have motivated for ongoing engagement in the study. | 30 | 14 | 30 | 100,0 | 2 | 6,7 | rather facilitator |
